# Superior canal dehiscence syndrome alters kinematics of head and postural control during standing

**DOI:** 10.1038/s41598-026-63082-1

**Published:** 2026-08-08

**Authors:** Raabeae Aryan, Jennifer L. Millar, John P. Carey, Michael C. Schubert, Kathleen E. Cullen

**Affiliations:** 1 Department of Biomedical Engineering, Johns Hopkins University School of Medicine, Baltimore, MD USA; 2 Department of Physical Medicine and Rehabilitation, Johns Hopkins University School of Medicine, Baltimore, MD USA; 3 Department of Otolaryngology-Head and Neck Surgery, Johns Hopkins University School of Medicine, Baltimore, MD USA; 4 Department of Neuroscience, Johns Hopkins University School of Medicine, Baltimore, MD USA; 5 Kavli Neuroscience Discovery Institute, Johns Hopkins University, Baltimore, MD USA

**Keywords:** Superior canal dehiscence syndrome, Vestibular system, Quiet standing, Balance, Health care, Medical research, Neurology, Neuroscience

## Abstract

Superior canal dehiscence syndrome (SCDS) is a peripheral vestibular disorder caused by a defect in the bony roof of the superior semicircular canal that alters inner-ear mechanics and produces abnormal vestibular stimulation and imbalance. Although postural instability is a prominent complaint in SCDS, how this disorder alters the kinematics of standing balance remains unclear. Here, we quantified how unilateral SCDS affects head and postural control during quiet standing using wearable inertial measurement units (IMUs). Angular velocities and linear accelerations were recorded from head and waist IMUs, enabling measurement of six-dimensional kinematics during seven standing balance tasks that systematically varied visual input and surface compliance. These measures revealed greater range and variability of head and waist motion in individuals with SCDS than in controls, indicating reduced stability during quiet standing. Group differences were greatest when visual and somatosensory feedback were degraded (e.g., standing on a foam surface with eyes closed), conditions that increase reliance on vestibular cues. Between-group differences were most consistently observed in pitch and roll kinematics, with increased yaw variability in several tasks. Variability in yaw head velocity correlated with oscillopsia and persistent postural-perceptual dizziness, whereas variability of waist vertical acceleration and yaw velocity mainly correlated with greater contra-lesional amplitude of cervical vestibular myogenic potential and impaired gait function, respectively. Global kinematic scores derived from IMU data significantly differentiated individuals with SCDS from healthy controls. Together, these findings demonstrate that unilateral SCDS produces multiplanar balance impairments, and that wearable kinematic analysis may provide a sensitive approach for quantifying postural instability and identifying underlying mechanisms of balance difficulties, complementing current standard functional balance assessments. In addition, our findings have the potential to inform future balance rehabilitation and fall prevention research in individuals with SCDS.

**Supplementary Information:**

The online version contains supplementary material available at https://doi.org/10.1038/s41598-026-63082-1.

## Introduction

Maintaining upright balance depends on the seamless integration of visual, proprioceptive, and vestibular inputs^1,2^. The vestibular system plays a unique role in detecting head motion and spatial orientation, enabling rapid reflexes that stabilize gaze and posture^3–6^. When vestibular function is compromised, postural stability declines and the risk of falls increases. Indeed, more than one-third of U.S. adults experience vestibular dysfunction^7^, and these individuals exhibit a markedly higher prevalence of falls^8^. Given this substantial impact, sensitive and precise assessment of balance is critical for rehabilitation and fall prevention strategies, particularly for disorders in which vestibular deficits are the primary driver of instability.

Disorders affecting individual semicircular canals provide a valuable framework for linking specific vestibular deficits to impairments in balance control. One such disorder, superior canal dehiscence (SCD), is a structural defect in which part of the bony roof overlying the superior semicircular canal is absent^9^. This “third mobile window” alters the transmission of sound and pressure within the inner ear, producing a distinctive combination of auditory and vestibular symptoms collectively referred to as superior canal dehiscence syndrome (SCDS)^10,11^. Auditory features include autophony, pulsatile tinnitus, bone-conduction hyperacusis, and low-frequency air–bone gaps, while vestibular features often include sound- or pressure-induced vertigo, oscillopsia, and chronic imbalance^11–13^. Mechanistically, the dehiscence alters the biomechanics of the superior semicircular canal, producing abnormal stimulation and reducing the reliability of vestibular signals from that canal. Such changes are expected to compromise postural control, particularly when visual or somatosensory cues are reduced. However, the extent to which these biomechanical changes translate into measurable alterations in postural kinematics remains unclear.

Despite the clear potential for SCDS to impair postural stability, this functional consequence remains poorly characterized. To our knowledge, only one prior study has assessed balance in this population, using standard bedside and functional assessments such as the modified Clinical Test of Sensory Integration on Balance, Dynamic Gait Index, Five Times Sit-to-Stand Test, and gait speed^14^. While valuable for identifying gross impairments and estimating fall risk, these measures provide limited insight into the underlying movement strategies or segmental coordination patterns used to maintain stability. In contrast, wearable inertial measurement units (IMUs) enable precise, multi-axis quantification of angular and linear motion in individual body segments, such as the head and trunk, throughout a balance task. This approach allows direct assessment of intersegmental coordination and dynamic postural strategies, revealing subtle deficits that may not manifest in functional scores but could nonetheless contribute to instability in daily life.

Here, we used wearable inertial measurement units (IMUs) to quantify multi-axis motion of individual body segments, providing six degrees of freedom (6DoF) measurements of rotational and translational kinematics. Because the superior semicircular canal primarily senses pitch and roll rotations, we expected the largest kinematic changes along these axes. These differences were expected to be most evident when visual or somatosensory cues were reduced. As a secondary objective, we examined correlations between kinematic measures and clinical measures of vestibular and balance function. By integrating high-resolution kinematic analysis with established clinical assessments, this work defines the specific balance deficits associated with SCDS and may provide a framework for future studies aimed at targeted rehabilitation and fall-prevention strategies.

## Methods

### Participants

From February 2022 to November 2023 we recruited 16 individuals with superior canal dehiscence (mean age ± SD = 46.8 ± 11.2) who were diagnosed by a neurotologist as having unilateral SCD symptoms (unilateral SCDS) based on the criteria explained in the consensus document from the Bárány Society International Classification of Vestibular Disorders^11^ (see Supplementary Tables 1 and 2 for clinical characteristics). Of those individuals, 8 had unilateral bony dehiscence, whereas the remaining participants had bilateral bony defects but unilateral symptoms consistent with unilateral SCDS. In these cases, the second side represented anatomical SCD without bilateral symptomatic SCDS. Clinical characteristics of these anatomical subgroups are summarized in Supplementary Table 3. Patients were not included if they had signs or symptoms suggestive of bilateral SCDS, previously received SCD repair, had other otologic conditions, or undergone other surgical procedures on either ear. Sixteen healthy age- and sex-matched participants (mean age ± SD = 47.3 ± 11.0) with no history of neurologic or otologic conditions were also recruited as the control group. For both groups, individuals were ineligible to participate if they had any acute and/or medically unmanaged migraine, were unable to ambulate independently, or had any other pathologies impacting their mobility and postural stability. This study was approved by the Institutional Review Board of Johns Hopkins University School of Medicine (IRB00246479) and was conducted in accordance with institutional guidelines for safe and ethical research in human participants. Written informed consent was obtained from all study participants before the start of the assessments.

### Balance assessment procedures and kinematic measurements

Participants were asked to maintain standing balance for 30 s under seven different quiet standing conditions, while their head and postural kinematics were recorded using two inertial measurement units (IMUs; Shimmer3, Shimmer Research, Dublin, Ireland). IMUs were affixed to the back of the head and at the waist level (between L4-S1), respectively. IMUs were synchronized at the beginning of each session using the ConsensysPro software (Shimmer Research, Dublin, Ireland). Each IMU recorded three-dimensional linear accelerations along the antero-posterior, mediolateral, and vertical axes (range set at ± 8 g), and angular velocities in the yaw, pitch, and roll planes (set at ± 2000 deg/s). Kinematic data were sampled at 500 Hz and stored on the built-in microSD cards for offline processing. Data were low-pass filtered at 25 Hz using a zero-phase 4^th^ order Butterworth filter. Linear acceleration signals were demeaned by subtracting the average acceleration value for each axis over the analyzed trial, thereby removing a constant offset associated with sensor orientation and the time-averaged acceleration signal. Because the linear acceleration features reported here were range and standard deviation, this constant-offset subtraction does not affect the reported kinematic values. Empirical static rotation matrices, determined according to the placement of each IMU, were then applied to align head and waist IMU signals to a common earth-referenced coordinate system. No additional processing was performed to separate gravity-related acceleration from transient/dynamic acceleration components.

To prevent falls and ensure safety, at least one researcher stood beside participants during each test. Rest breaks were provided as requested. Participants performed the following tasks (Table 1, and Supplementary Fig. 1): (1) standing on a firm surface (ground) with eyes open, arms folded across chest, and feet together; (2) standing on a firm surface with eyes closed, arms folded, and feet together; (3) standing on a high density foam cushion (a rectangular 5 cm × 36 cm × 45 cm balance foam pad, ArmaSport by Armacell) with eyes open, arms folded, and feet together; (4) standing on a foam cushion with eyes closed, arms folded, and feet together; (5) standing on a firm surface with eyes open, arms folded, and feet in tandem; (6) standing on a firm surface with eyes closed, arms folded, and feet in tandem; (7) standing with one foot on the ground and the other on a foam cup (without crushing the cup) for 15 s, then switching feet and repeating for an additional 15 s (i.e., single-leg foam-cup stance balance task). During the tandem stance conditions, participants were instructed to place their feet heel-to-toe, with their dominant foot placed in front. To determine the dominant foot, participants were asked which foot they would use to kick a ball if a ball were rolled toward them; that foot was then considered the dominant foot.

**Table 1:** Quiet standing balance tests performed in this study.

Tasks	Vision	Stance support surface
Standing with feet together	Eyes open	Firm surface
Standing with feet together	Eyes closed	Firm surface
Standing with feet together	Eyes open	Foam cushion
Standing with feet together	Eyes closed	Foam cushion
Standing with feet in tandem	Eyes open	Firm surface
Standing with feet in tandem	Eyes closed	Firm surface
Standing with 1 foot on the ground, and 1 foot on a foam cup	Eyes open	Firm surface (stance foot)

Due to technical issues during the experiments, we could not use head IMU data from one SCDS participant. All participants completed the balance protocol, except for a small number of missing trials for the following reasons: (a) One SCDS participant was unable to maintain balance without external support during tasks of tandem stance with eyes closed, and single-leg foam-cup stance (tasks 6 and 7). Therefore, for this participant no kinematic data were recorded during these trials, and completion times for both trials were recorded as zero; (b) Due to scheduling constraints, single-leg foam-cup stance (task 7) was not assessed in another SCDS participant, and standing on firm surface with eyes open (task 1) was not assessed in four control participants; thus, for these individuals, no completion time or kinematic data were recorded during the specified trials (Supplementary Table 4 reports the exact size of both groups for each balance task). Uncrossing the arms for support and moving the feet out of the test position or taking a step to regain balance, if any, were further detected during signal processing and segmentation with the help of additional sensors on the wrist or ankle. In such cases, participants were allowed to return to the test position, if willing, and continue within their original 30-s trial duration. However, signal segments corresponding to these events were excluded from further processing.

Finally, we computed the following measures to quantify head and waist movements during each quiet standing balance test: (a) the duration for which participants were able to maintain their balance, (b) the range of linear accelerations and angular velocities during the test period, defined as the difference between the maximum and minimum values of each kinematic measure during a given task, i.e., the peak-to-peak amplitude; and (c) the standard deviation (SD) of linear accelerations and angular velocities during the test period. Range and SD were used as complementary descriptors of the magnitude and variability of postural motion, respectively.

In addition, we calculated kinematic scores to evaluate whether a composite score derived by aggregating multiple kinematic measures across balance tests could effectively distinguish between the SCDS and control groups. To compute the kinematic score, each individual kinematic measure (i.e., range and SD of angular velocities and linear accelerations) was linearly normalized using the mean and SD calculated from the combined population of healthy control and SCDS participants for that measure and task. Specifically, the combined-population mean was mapped to a normalized score of 50, and the combined-population SD was mapped to 25; the resulting linear transformation, including both scaling and offset factors, was then applied identically to healthy control and SCDS participant values. Values outside the mean ± 2 SD range were projected to the upper or lower bounds of the 0–100 scale. The normalized values were then averaged across the balance tasks that were common to both IMUs and showed the most consistent between-group differences across kinematic measures^15,16^.

### Clinical assessments

To investigate the relationship between kinematic measures and clinical outcomes, the following physiological and functional measures and questionnaires were administered:

**Physiological vestibular measures**:

Video Head Impulse Test (reviewed in^17^): The vHIT was used to assess the effectiveness of the vestibular semicircular canals in response to passive head rotations. Particularly, vHIT was administered to evaluate vestibulo-ocular reflex (VOR) gains for all canal planes in both ears. In a well-lit room, participants were seated on a plinth or chair at a 1-meter distance from a fixed visual target on the wall, while wearing the vHIT headset (ICS, Otometrics/Natus Medical Incorporated, Denmark). Passive head rotations were delivered by the examiner along the three planes parallel to the three pairs of semicircular canals, namely, horizontal, right anterior-left posterior (RALP), and left anterior-right posterior (LARP) planes.

Vestibular Evoked Myogenic Potentials^18^: VEMP testing was used to evaluate otolith organ function. Amplitudes of cVEMP (rectified cervical VEMP amplitude, to test saccular function) and oVEMP (ocular VEMP amplitude, to test utricular function) were extracted, when available, from SCDS participants’ clinical records. VEMP asymmetry ratios were calculated at the participant level and reported as percentages. Specifically, oVEMP asymmetry was calculated as the absolute value of 100 × (Amplitude Right − Amplitude Left)/(Amplitude Right + Amplitude Left), and cVEMP asymmetry was calculated as the absolute value of 100 × (rectified Amplitude Right − rectified Amplitude Left)/(rectified Amplitude Right + rectified Amplitude Left).

**Functional measures**:

Dynamic Visual Acuity test^19,20^: The DVA was used to examine the efficacy of the VOR in response to active sinusoidal horizontal and vertical head rotations. Participants were seated on a chair at a 2-m distance from a tablet screen (Samsung Galaxy Pro, Seoul, South Korea) while wearing a head-mounted inertial sensor (Xsens Technologies, Enschede, Netherlands). A set of ten optotypes (i.e., C D H K N O S R V Z) were presented one at a time in a random order on the tablet screen. Participants fixated on the screen to identify the correct displayed letters during the following five conditions: (1) head stationary (static visual acuity); (2) sinusoidal horizontal head rotations with the letters presented during rightward head rotations; (3) sinusoidal horizontal head rotations with the letters presented during leftward head rotations; (4) sinusoidal vertical head rotations with the letters presented during upward head rotations; and (5) sinusoidal vertical head rotations with the letters presented during downward head rotations. These active head movements were participant-paced, and their frequency and amplitude were not prescribed. During conditions 2–5 of the DVA assessment, the software displayed a new letter on the monitor once a head rotational velocity greater than 120 deg/s was detected. In cases where the head movements were not fast enough to generate an optotype, participants were instructed to increase the speed of their head movements. DVA scores were reported as corrected LogMAR scores (logarithm of the minimum angle of resolution), calculated by subtracting the dynamic scores from the static score. DVA scores for rightward and leftward horizontal head rotations were subsequently organized as ipsi-lesional and contra-lesional, corresponding to yaw head rotations to the symptomatic and non-symptomatic sides, respectively.

Gait Speed: Normal gait speed was evaluated over a 10-meter walkway. Participants were instructed to walk at their comfortable walking speed and performed two trials of this test. After completing the first trial, they repeated the task, starting from the opposite end of the hallway. Gait speed tests were completed in a hospital hallway, with participants starting and ending approximately 1.3–3.8 m before and after (depending on the starting end of the hallway) the timed 10-m part of the test to allow for acceleration and deceleration. Average gait speed was calculated from the two trials.

Single-task Timed Up and Go test (TUG)^21^: Participants started in a seated position in an armchair. On the go command, they stood up, walked three meters at their normal speed, turned around a cone placed 3 meters from the chair, walked back to the chair, and sat down. The TUG test was performed twice: once with an ipsi-lesional turn and once with a contra-lesional turn. The average TUG time was calculated from the two trials for further analysis.

Cognitive Dual-task Timed Up and Go test (TUG.Cog): In this test, participants performed the same task as in single-task TUG while concurrently counting backward by three. Similarly, participants completed one trial with an ipsi-lesional turn and one trial with a contra-lesional turn. The average TUG.Cog time was calculated from the two trials for further analysis.

Functional Gait Assessment scale^22^: The FGA scale was used to evaluate dynamic postural stability during walking tasks, which consists ten items as follows: (1) gait on a level surface, (2) change in gait speed, (3) gait with horizontal head turns, (4) gait with vertical head turns, (5) gait and pivot turn, (6) gait while stepping over an obstacle, (7) gait with narrow base of support, (8) gait with eyes closed, (9) ambulating backwards, and (10) steps. Performance during each item was rated by an examiner, with scores ranging from 0 (unable/severely impaired) to 3 points (normal).

**Participant-reported outcome measures:** To assess self-perceived balance confidence, anxiety level, headache impact, impact of oscillopsia, severity of postural dizziness, and dizziness-induced disability during activities of daily living, participants completed the Activities-specific Balance Confidence (ABC)^23^, Beck Anxiety Inventory (BAI)^24^, Headache Impact Test (HIT-6)^25^, Oscillopsia Functional Impact (OFI)^26^, Niigata Persistent Postural-Perceptual Dizziness (PPPD)^27^, and the Dizziness Handicap Inventory (DHI)^28^ questionnaires, respectively. Autophony, the pathological ability to hear individuals’ own voice or internal bodily sounds, was evaluated using the Autophony Index questionnaire, which was either administrated by the research team or obtained from medical records when completed concurrently by the clinical team.

### Statistical analysis

Comparisons between healthy controls and the unilateral SCDS group were performed using a non-parametric independent permutation test (re-randomization)^29^ with 100,000 randomized rearrangements of data points. To evaluate the correlations between head and waist kinematic and clinical measures of the SCDS group, data normality was first tested by using the Shapiro–Wilk test; accordingly, either Spearman’s or Pearson’s correlation coefficients were computed. To identify consistent correlation trends between the kinematic and clinical measures, we assessed whether correlations across balance tasks: (a) were statistically significant (*p* < 0.05), and (b) showed the same sign (i.e., correlations were consistently positive or negative across multiple tasks). All statistical analyses and data processing were performed using custom-written MATLAB codes (2021b, The MathWorks Inc., Natick, MA, USA). Statistical significance level was set at α = 0.05, and values were reported as mean ± 1 SD. Given the exploratory nature of this study, the relatively small sample size, and our goal of identifying candidate kinematic features and clinical associations for future hypothesis-driven work, *p* values were not corrected for multiple comparisons or correlations and should therefore be interpreted as exploratory.

## Results

Demographic and clinical characteristics of the participants are summarized in Supplementary Tables 1 and 2. Supplementary Fig. 2 highlights the differences between healthy controls and SCDS group in balance confidence levels, postural perceptual dizziness, and functional impact of oscillopsia (*p* < 0.01). Supplementary Fig. 3 provides descriptive clinical context for vHIT-induced VOR gains, using the average of both sides in healthy controls for comparison with ipsi- and contra-lesional canals in the SCDS group. In this descriptive comparison, ipsi-lesional anterior canal VOR gain was lower in the SCDS group than the healthy-control average (*p* < 0.01).

### Head and waist kinematics are altered in unilateral SCDS, particularly under reduced sensory conditions

**Fig. 1:**
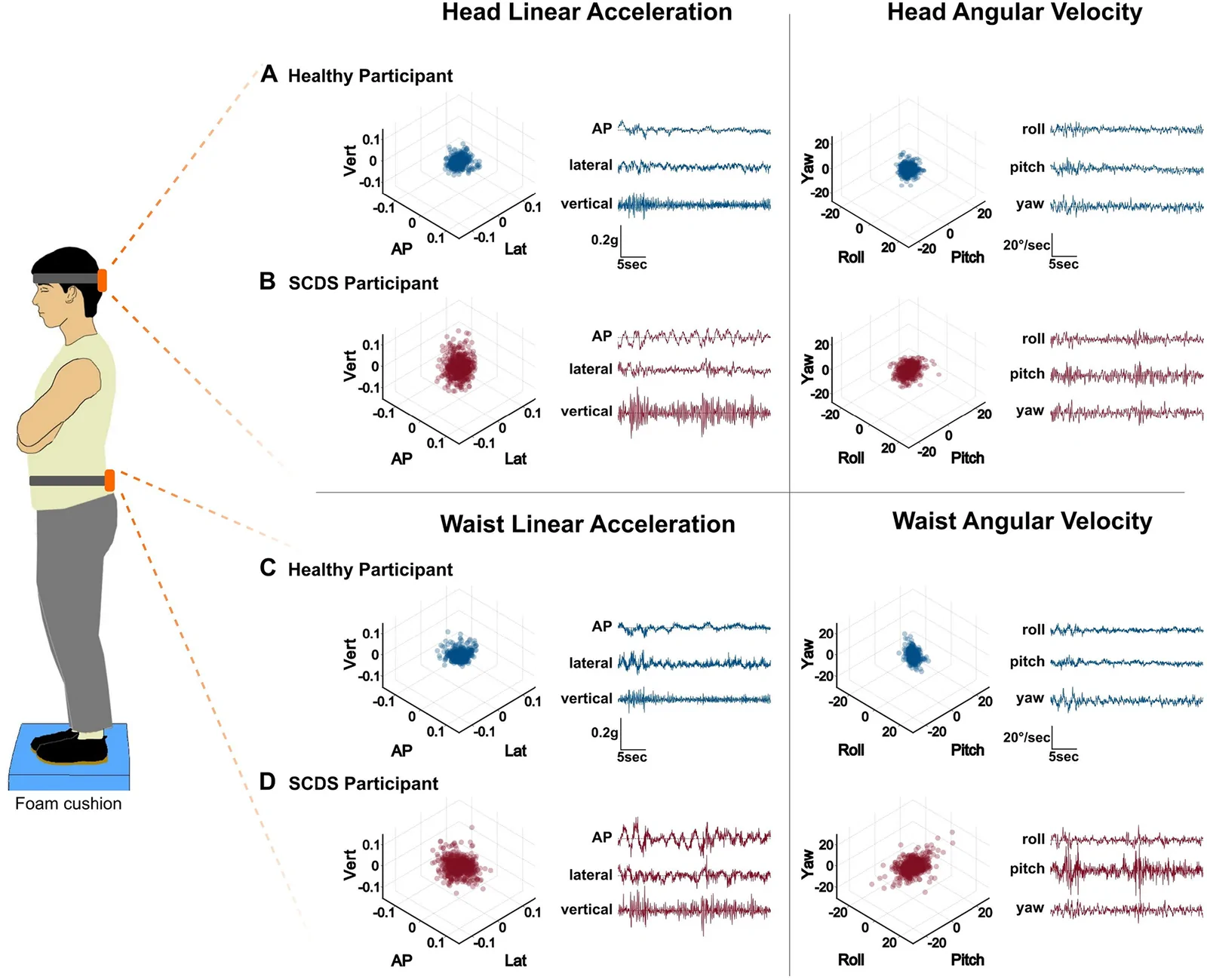
Examples of raw linear accelerations (left panels) and angular velocities (right panels) extracted from the head (top panels) and waist (bottom panels) IMUs, recorded from a representative healthy participant (male, age 43 years; blue traces and scatterplots in (**A**) and (**C**)) and a representative individual with SCDS (male, age 46 years; red traces and scatterplots in (**B**) and (**D**)) during standing on a foam cushion with eyes closed. Lat: lateral. Vert: vertical. Fore-aft: antero-posterior. SCDS: superior canal dehiscence syndrome.

To determine whether unilateral superior canal dehiscence syndrome (SCDS) alters postural kinematics during quiet standing, we compared head and waist kinematics between sixteen individuals with unilateral SCDS and sixteen age- and sex-matched healthy controls while they performed seven quiet standing balance tasks that systematically varied visual input and base of support (Table 1). Figure 1 illustrates representative triaxial angular velocity and linear acceleration recordings from the head and waist IMUs of a typical healthy control (blue traces) and an individual with left-sided SCDS (red traces) during one of the most challenging balance conditions, standing on a foam pad with eyes closed for 30 s. Compared with the control participant, the individual with SCDS exhibited larger excursions in both linear and rotational head and waist kinematics, illustrating increased movement variability during this condition.

**Fig. 2:**
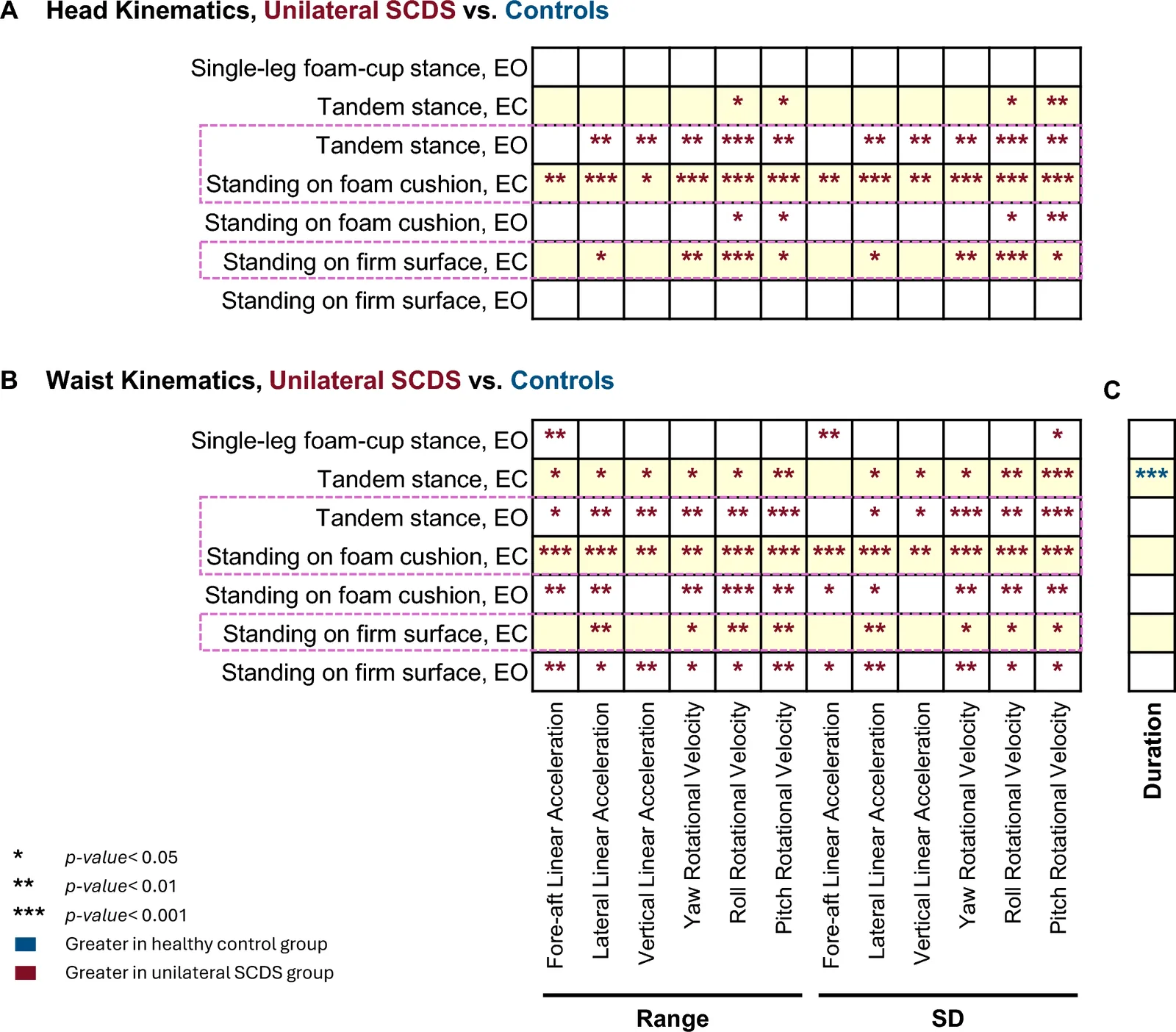
Between-group comparisons of angular velocities (i.e., pitch, roll, yaw) and linear accelerations (i.e., vertical, lateral, fore-aft) computed from the (**A**) head, and (**B**) waist IMUs during all seven standing balance tasks. (**C**) Between-group comparison of the average duration for which participants were able to maintain their standing balance in each balance task condition. Blue asterisks indicate statistically significant differences with greater values for the healthy control group, and red asterisks indicate statistically significant differences with greater values for the SCDS group. Light yellow rows specify the balance tests with eyes closed. Magenta dotted boxes indicate the balance tests which were used to calculate the combined global kinematic score. Maximum test duration: 30 s. SCDS: superior canal dehiscence syndrome. EO: eyes open. EC: eyes closed. *p < 0.05, **p < 0.01, and ***p < 0.001. SD: standard deviation. See Supplementary Table 4 for the exact size of both groups for each balance task.

We next quantified group-level differences in kinematic measures across all balance tasks to determine whether this pattern generalized across participants and task conditions. Figure 2A summarizes between-group differences in kinematic measures extracted from the head IMU across all seven balance tasks. Numerical values for the range of linear accelerations and angular velocities are provided in Supplementary Tables 5 and 6, and corresponding values for variability (SD) are provided in Supplementary Tables 7 and 8. Head kinematics in the SCDS group were significantly greater than those of healthy controls in five of seven tasks. The greatest number of significant between-group differences occurred during standing on a foam pad with eyes closed, tandem stance with eyes open, and standing on a firm surface with eyes closed. In particular, standing on a foam pad with eyes closed revealed significant differences across all rotational and linear head kinematic measures (*p* < 0.05). In contrast, no significant between-group differences were observed for any kinematic measures during the single-leg foam-cup stance and standing on a firm surface with eyes open tasks (*p* > 0.05). The dashed magenta boxes in Fig. 2A and B indicate the three balance tasks used to calculate the combined kinematic score, because these tasks were common to both the head and waist IMUs and showed significant between-group differences across a greater number of kinematic measures.

To determine which head kinematic metrics contributed most strongly to these between-group differences, we examined rotational and linear components separately (Fig. 2A). Head rotational kinematics showed the most consistent between-group differences across balance tasks. Across head kinematic measures, angular velocity metrics were the most informative. The range and variability (SD) of roll and pitch angular head velocities differed significantly between groups in five out of seven tasks, while yaw angular velocity range and variability differed in three out of seven tasks (*p* < 0.05). Thus, both range and variability in all three angular axes emerged as kinematic measures consistently associated with group differences across tasks, indicating altered head rotational stability in unilateral SCDS. In contrast, the range and variability of lateral, vertical, and antero-posterior linear head accelerations differed significantly between groups in three, two, and one of the seven quiet standing balance tasks, respectively (*p* < 0.05).

Between-group differences were even more pronounced for waist kinematics measured near the body’s center of mass (Fig. 2B). Numerical values for the range of linear accelerations and angular velocities computed from the waist IMU are presented in Supplementary Tables 9 and 10, and corresponding values for variability (SD) are presented in Supplementary Tables 11 and 12. Compared with healthy controls, individuals with unilateral SCDS exhibited significantly greater range and variability of waist kinematic measures. Specifically, analysis of angular waist velocities revealed that variability (SD) of pitch angular velocity differed significantly between groups in all balance tasks (*p* < 0.05), while variability of roll and yaw velocities as well as the range of all three angular velocities differed significantly in six of seven tasks (*p* < 0.05).

Analysis of waist linear acceleration measures revealed a similar pattern of between-group differences. The range of antero-posterior and lateral linear accelerations differed significantly between groups in six of seven balance tasks (*p* < 0.05), whereas the range of vertical waist acceleration differed significantly in four of seven tasks (*p* < 0.05). In addition, variability of lateral, antero-posterior, and vertical linear waist accelerations showed significant between-group differences in six, four, and three of seven balance tasks, respectively (*p* < 0.05). Notably, as observed for head kinematics, the most robust between-group differences occurred during standing on a foam pad with eyes closed (*p* < 0.01), while fewer significant differences were observed during the single-leg foam-cup stance balance task.

Thus, across measures, angular velocity metrics revealed more consistent between-group differences than linear acceleration measures. Moreover, waist kinematics differed across a greater number of balance tasks than head kinematics, indicating that motion recorded at the waist level, which approximates body center of mass motion during quiet standing, provided a more sensitive marker of altered postural control in unilateral SCDS.

To determine whether group differences in head and waist kinematics were driven by differences in balance duration, we next assessed task completion time (Fig. 2C). Mean and SD of task-specific completion times are presented in Supplementary Table 13. Figure 2C compares the duration for which participants in each group were able to maintain balance within the specified task conditions. A significant between-group difference was observed only during tandem stance with eyes closed, for which healthy controls maintained balance for a significantly longer period than individuals with unilateral SCDS (*p* < 0.001). No significant differences in balance duration were observed for the remaining balance tasks, indicating that group differences in kinematic measures generally occurred even when task completion time was comparable.

### Relationship between head and waist kinematics during quiet standing

To further examine segmental coordination during standing balance, we performed a supplementary correlation analysis between head and waist angular velocity measures in the SCDS and control groups across balance tasks (Supplementary Figs. 4–7). Overall, head and waist angular velocity measures were correlated in both groups, but the pattern of coupling differed between individuals with SCDS and healthy controls. During standing on a firm surface with eyes open, individuals with SCDS showed a greater number of significant correlations between head and waist angular kinematics than healthy controls, and these correlations became more pronounced during the eyes-closed condition (Supplementary Fig. 4A and B). During standing on foam and tandem stance, SCDS participants again showed more significant head–waist correlations than controls when visual information was available (Supplementary Figs. 5A and 6A). However, when visual input was removed during these more challenging conditions, SCDS participants showed fewer significant head–waist correlations than controls (Supplementary Figs. 5B and 6B). During single-leg foam-cup stance with eyes open, individuals with SCDS also showed significant correlations across a greater number of angular kinematic measures than healthy controls (Supplementary Fig. 7).

Together, these exploratory findings suggest that unilateral SCDS alters head–waist coupling during quiet standing. Specifically, head and waist movements appear more strongly coupled in SCDS when visual information is available, whereas under more challenging eyes-closed conditions with reduced somatosensory reliability, this coupling may become less consistent. This task-dependent pattern may reflect altered intersegmental coordination during balance control, although future studies will be needed to test this possibility directly.

### Kinematic measures during standing balance correlate with symptom severity and gait impairment

**Fig. 3:**
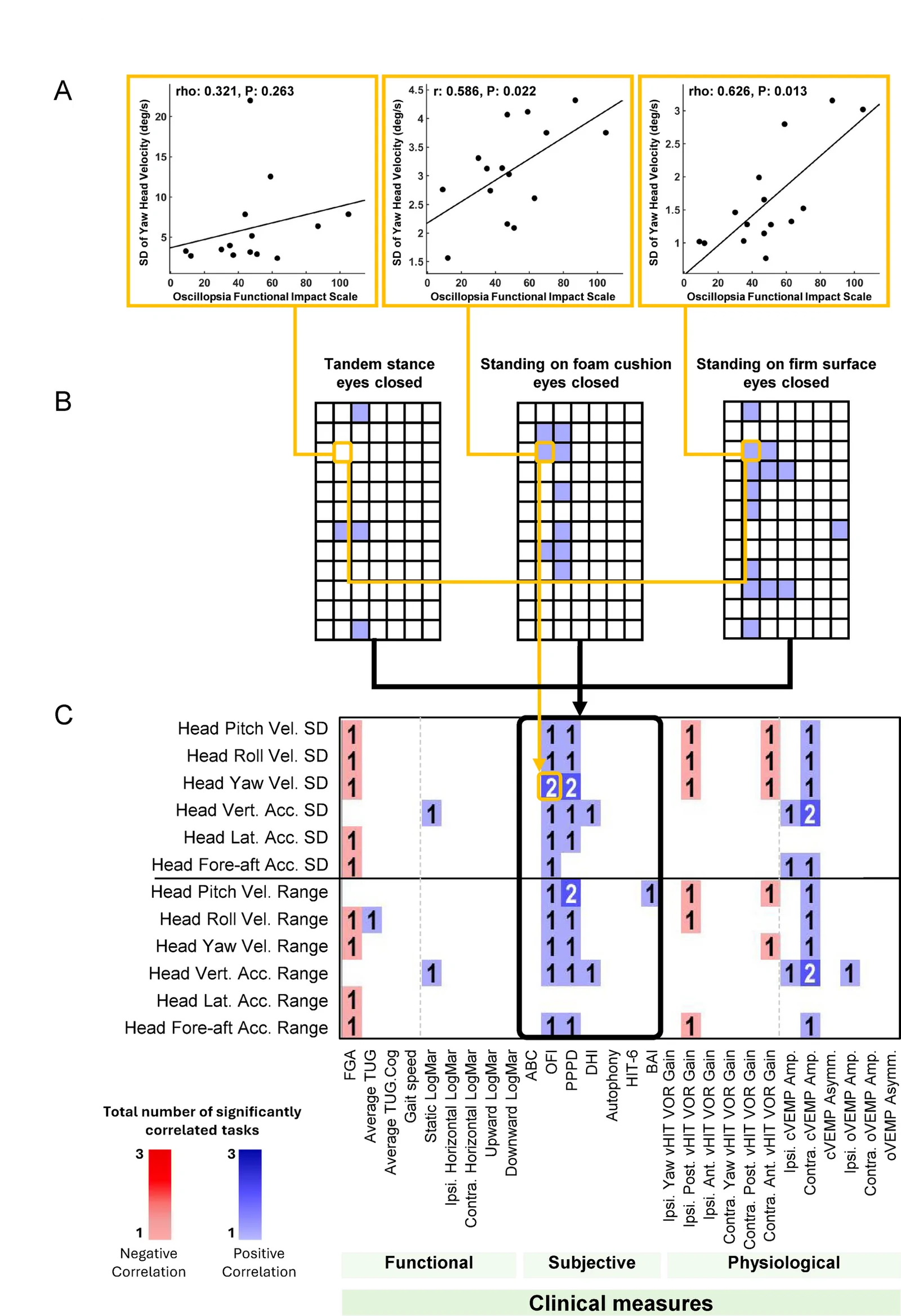
Correlations between the SCDS group’s head kinematics (shown on the y-axis) during three balance tasks with eyes closed (i.e., standing on a firm surface with eyes closed, standing on a foam cushion with eyes closed, tandem stance with eyes closed) and their clinical measures shown on the x-axis. (A) The example scatterplots illustrate the status of correlations between the SD of the yaw head velocity and the oscillopsia functional impact score (OFI) during each task with eyes closed, separately. (B) The examples show that the SD of yaw head velocity is significantly correlated with the OFI score only during tasks of standing on a foam cushion with eyes closed and standing on a firm surface with eyes closed. (**C**) Correlation map between head kinematic measures (y-axis; i.e., pitch, roll, yaw angular velocities, and vertical, lateral, antero-posterior linear accelerations) during three balance tasks performed with eyes closed and the clinical measures (x-axis). Blue squares represent statistically significant positive correlations, and red squares represent statistically significant negative correlations. Brightness and the numbers in each cell of the map indicate the total number of tasks (out of 3) in which a significant correlation was observed between the kinematic and clinical measures (*p* value < 0.05). SD: standard deviation. Acc: acceleration. Vel: velocity. Ipsi: ipsi-lesional. Contra: contra-lesional. FGA: Functional Gait Assessment scale. TUG: Timed Up and Go test. TUG.Cog: cognitive (dual task) Timed Up and Go test. Lat: lateral. Vert: vertical. Fore-aft: antero-posterior. OFI: Oscillopsia Functional Impact questionnaire. DHI: Dizziness Handicap Inventory questionnaire. BAI: Beck Anxiety Index questionnaire. PPPD: Niigata Persistent Postural-Perceptual Dizziness questionnaire. HIT-6: Headache Impact Test questionnaire. Autophony: Autophony Index questionnaire. ABC: Activities-specific Balance Confidence questionnaire. VOR: Vestibulo-Ocular Reflex. vHIT: Video Head Impulse Test. Post: Posterior. Ant: Anterior. cVEMP: Cervical Vestibular Evoked Myogenic Potential. oVEMP: Ocular Vestibular Evoked Myogenic Potential. Amp: amplitude. Asymm: asymmetry ratio. See Supplementary Table 4 for the exact size of both groups for each balance task.

**Fig. 4:**
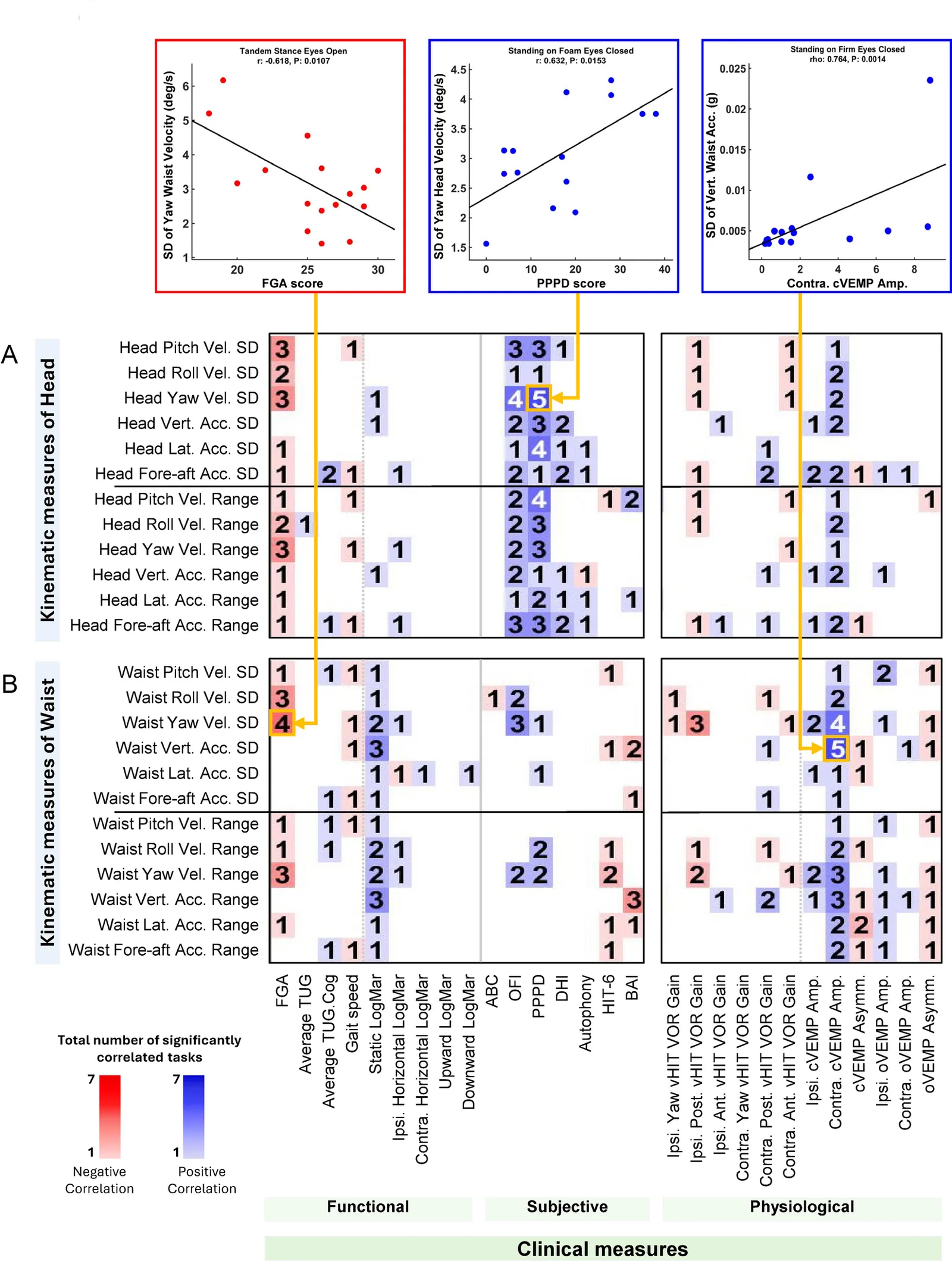
Maps of correlations between the clinical measures of individuals with SCDS (x-axis; functional, subjective, and physiological) and their kinematic measures (y-axis) during all seven standing balance tasks, calculated for (**A**) head, and (**B**) waist IMUs. Blue and red squares reflect positive and negative correlations, respectively. Brightness and the numbers in each cell indicate the total number of tasks (out of 7 tasks) for which a significant correlation between the kinematic and clinical measures was observed (*p* < 0.05). The top three scatterplots show examples of statistically significant correlations between (from left to right): the FGA score and SD of yaw waist velocity during tandem stance with eyes open (significant negative correlation; highlighted in red), the PPPD score and SD of yaw head velocity during standing on foam with eyes closed (significant positive correlation; highlighted in blue), and between the cVEMP amplitude of contra-lesional side and SD of waist vertical acceleration during standing on firm surface with eyes closed (significant positive correlation; highlighted in blue). SD: standard deviation. Acc: acceleration. Vel: velocity. Ipsi: ipsi-lesional. Contra: contra-lesional. FGA: Functional Gait Assessment scale. TUG: Timed Up and Go test. TUG.Cog: cognitive (dual task) Timed Up and Go test. Lat: lateral. Vert: vertical. Fore-aft: antero-posterior. OFI: Oscillopsia Functional Impact questionnaire. DHI: Dizziness Handicap Inventory questionnaire. BAI: Beck Anxiety Index questionnaire. PPPD: Niigata Persistent Postural-Perceptual Dizziness questionnaire. HIT-6: Headache Impact Test questionnaire. Autophony: Autophony Index questionnaire. ABC: Activities-specific Balance Confidence questionnaire. VOR: Vestibulo-Ocular Reflex. vHIT: Video Head Impulse Test. Post: Posterior. Ant: Anterior. cVEMP: Cervical Vestibular Evoked Myogenic Potential. oVEMP: Ocular Vestibular Evoked Myogenic Potential. Amp: amplitude. Asymm: asymmetry ratio. See Supplementary Table 4 for the exact size of both groups during each balance task.

To assess the functional relevance of the observed kinematic alterations, we next examined whether head and waist kinematic measures during standing balance were associated with clinical measures of symptom severity and gait performance (Figs. 3 and 4). Because removing visual input increases reliance on vestibular signals during postural control, we first focused on the three balance tasks performed with eyes closed to determine whether kinematic measures under these vestibular-demanding conditions relate to clinical symptoms. Figure 3 illustrates the results from the three eyes-closed balance tasks: standing on foam with eyes closed, tandem stance with eyes closed, and standing on a firm surface with eyes closed. The standard deviation of yaw angular head velocity was significantly and positively correlated with both the Oscillopsia Functional Impact score and perceived postural dizziness in two of the three eyes-closed tasks (*p* < 0.05). Range and standard deviation of vertical head acceleration was also significantly and positively correlated with amplitude of contra-lesional cVEMP in two out of three eyes-closed tasks (*p* < 0.05). As summarized in the correlation map in Fig. 3C, greater variability of yaw head velocity was thus associated with more severe perception of dizziness and oscillopsia, while increased range and variability of vertical head acceleration was associated with larger cVEMP amplitude in the contra-lesional ear. In contrast, fewer significant correlations were observed between head kinematic measures during eyes-closed tasks and the functional or remaining physiological measures.

We next asked whether these relationships generalized beyond the eyes-closed conditions to the full set of standing balance tasks. Accordingly, we examined correlations across all seven balance tasks (Fig. 4). Across tasks, greater yaw head velocity variability was significantly correlated with perceived postural dizziness in five of seven tasks and with the Oscillopsia Functional Impact in four of seven tasks (Fig. 4A, *p* < 0.05), indicating that increased head motion variability during quiet standing is associated with greater symptom severity. Figure 4B further shows that, for waist kinematics, the most consistent correlations were observed between the standard deviation of waist vertical acceleration and amplitude of contra-lesional cVEMP, followed by the correlations between the standard deviation of yaw waist angular velocity with amplitude of contra-lesional cVEMP, as well as with total Functional Gait Assessment (FGA) score. Therefore, during standing balance, larger cVEMP amplitude on the contra-lesional side, in other words greater saccular responsiveness on the non-symptomatic side, was associated with greater variability of waist-level yaw velocity and vertical acceleration, whereas poorer gait performance was associated with greater waist-level yaw motion variability.

### Global kinematic scores derived from standing on foam with eyes closed differentiate individuals with SCDS from healthy controls

**Fig. 5:**
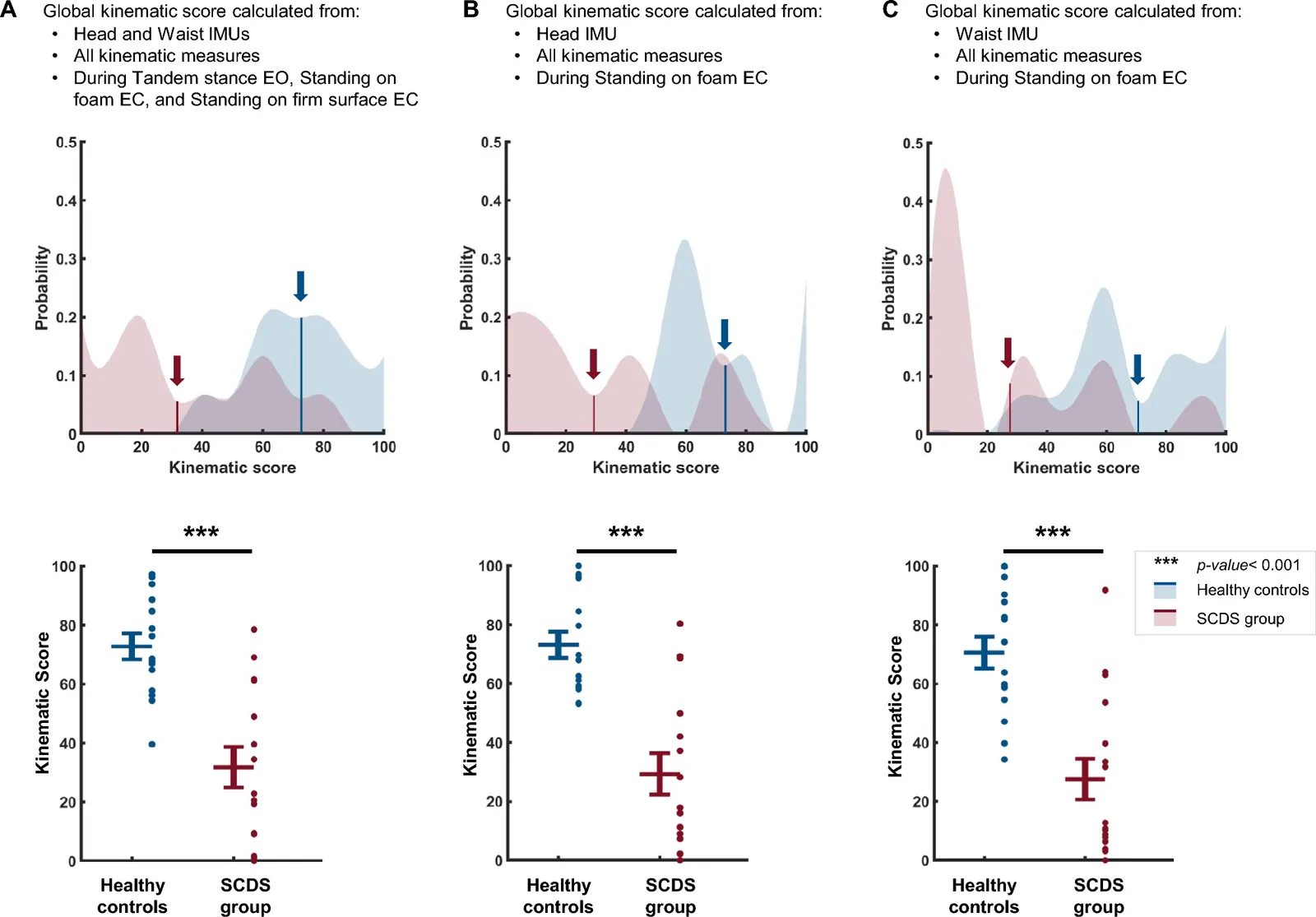
Differentiating between the healthy control group (blue) and SCDS (red) group using three global kinematic scores: (**A**) the global kinematic score calculated from both head and waist IMUs combined, using all kinematic measures during tasks of tandem stance with eyes open, standing on a foam cushion with eyes closed, and standing on a firm surface with eyes closed (calculated from 15 SCDS participants who had both head and waist IMU data and their controls); (**B**) the global kinematic score calculated from only the head IMU, using all kinematic measures during task of standing on a foam cushion with eyes closed (calculated from 15 SCDS participants who had head IMU data and their controls); and (**C**) global kinematic score calculated from only the waist IMU, using all kinematic measures during task of standing on a foam cushion with eyes closed (calculated from all SCDS participants and controls). Arrows in the top plots represent the average global kinematic scores for each group. Scatter dots represent individual kinematic scores. The vertical lines in the bottom plots represent ± 1 SEM (standard error of the mean). The shaded areas represent the probability distribution of the global kinematic scores for each group. EO: eyes open. EC: eyes closed. ***p < 0.001.

Finally, we asked whether the observed group differences could be summarized using a reduced set of balance tasks to generate a clinically useful composite metric. To address this, we computed global kinematic scores (see Methods) to test whether a composite measure based on tasks that consistently differed between groups could differentiate individuals with unilateral SCDS from healthy controls (Fig. 5; kinematic scores are presented in Supplementary Table 14).

As shown in Fig. 5A, a global score combining head and waist kinematics significantly separated individuals with unilateral SCDS from controls. This score was derived from all predefined linear acceleration and angular velocity measures analyzed in Fig. 2, rather than from a selected subset of axes or metrics. Specifically, it included the ranges and SDs obtained during the three tasks that were common to both the head and waist IMUs and that continued to show significant between-group differences across a greater number of kinematic measures (highlighted with dashed magenta boxes in Fig. 2A and B); namely: standing on foam with eyes closed, tandem stance with eyes open, and standing on a firm surface with eyes closed. We next asked whether similar discrimination could be achieved using a single task. Accordingly, additional kinematic scores were computed using head-only (Fig. 5B) and waist-only (Fig. 5C), using all kinematic measures obtained during standing on foam with eyes closed, which was the task that demonstrated the greatest number of significant between-group differences across both IMUs.

All three global kinematic scores significantly differentiated individuals with unilateral SCDS from healthy controls. Notably, scores computed from a single task using either the head or waist IMU alone demonstrated comparable discriminatory power to scores computed using both sensors across multiple tasks. Among these, the score derived from head kinematics during standing on foam with eyes closed demonstrated the strongest statistical separation between groups (Fig. 5B; Supplementary Table 14). To further assess individual-level separation between groups, we calculated the proportion of SCDS participants whose kinematic scores fell within or outside reference ranges defined by the healthy-control mean ± 1 SD and mean ± 2 SD (Supplementary Table 15). This analysis showed that the head-IMU score during standing on foam with eyes closed provided the clearest individual-level separation: only 20% and 33.3% of SCDS participants fell within the healthy-control ± 1 SD and ± 2 SD ranges, respectively, meaning that 80% and 66.7% fell outside these ranges.

## Discussion

Prior studies have reported that individuals with superior canal dehiscence syndrome (SCDS) experience chronic disequilibrium and vertigo^12,30^. However, it is not known whether dysfunction of a single symptomatic superior semicircular canal is sufficient to measurably disrupt head and body postural control during quiet standing. Here, we addressed this question using wearable inertial measurement units (IMUs) to quantify full six degrees of freedom (6DoF) kinematics—three-dimensional angular velocities (yaw, pitch, roll) and three-dimensional linear accelerations (antero-posterior, medio-lateral, vertical)—of both the head and waist-level across standing tasks that systematically manipulated visual and somatosensory reliability. Our results demonstrate that unilateral SCDS alters standing balance, producing increased range and variability of both rotational and translational motion. Importantly, these impairments were not confined to the anatomical plane of the superior canal (pitch), but instead manifested as consistent multiplanar instability, revealing that a focal alteration in semicircular canal biomechanics leads to distributed changes in multidimensional postural control.

Postural stability depends on the nervous system’s ability to estimate body orientation in space through the integration of vestibular, visual, and proprioceptive signals. During upright standing, the nervous system must continuously estimate body orientation and center of mass position relative to gravity and the base of support (reviewed in^31^ and^32^), a process that depends on the integration of proprioceptive, visual, and vestibular inputs^2,33^. Moreover, control of anterior–posterior and medio-lateral postural sway in standing relies on distinct ankle and hip mechanisms that vary with stance width and task demands^34,35^. For example, narrowing the base of support medio-laterally (such as in feet together or tandem stance) increases medio-lateral challenge, and potentially decreases the effective contribution of proprioceptive feedback^34^. Therefore, in the present study, given our focus on SCDS, we evaluated balance under conditions that systematically manipulated the availability and reliability of visual and somatosensory cues. Head- and waist-mounted IMUs were used to quantify segmental motion: subtle head movements engage the vestibular semicircular canals and otolith organs, waist sensors approximate postural and center of mass dynamics, and stabilization of both segments reflects the integration of vestibulospinal and multisensory inputs. By quantifying both rotational and translational motion across these segments, we moved beyond time-based task completion metrics to characterize the dynamic structure of balance control under altered vestibular reliability.

We hypothesized that kinematic changes would be greatest in pitch and roll, reflecting the sensitivity of the superior semicircular canal to rotations in these axes. Consistent with this prediction, group differences were most consistently observed in pitch and roll kinematics. However, yaw variability was also increased in several tasks, indicating that unilateral SCDS produces a pattern of multiplanar instability rather than a deficit confined to a single canal-aligned axis. This finding is informative from a systems perspective. Although superior canal dehiscence primarily alters biomechanics within a single canal plane, upright stance requires coordinated control of head-in-space, head-on-body, and trunk motion across three dimensions to maintain the center of mass within the base of support^2,36^. Vestibular signals contribute broadly to the control of axial and appendicular muscles during standing, allowing perturbations in one canal to influence postural motion across multiple axes^37,38^, reviewed in^39^. Thus, reduced reliability in signals from a single canal can alter central estimates of body motion and orientation that contribute to postural control^40^. Consistent with this framework, degraded canal signaling can influence central sensory integration processes that estimate body orientation and self-motion in three dimensions (reviewed in^41^ and^42^). In our recent locomotion study, individuals with SCDS showed changes in movement primarily in pitch and vertical motion—reflecting the dominant axes of motion during walking and consistent with compensatory strategies that may reduce stimulation of the affected canal^15^—whereas during quiet standing we observe more distributed, multiplanar instability.

The observation of increased yaw variability is especially informative in this context. Yaw rotations are central to heading control and visual–vestibular alignment during upright stance. Even small head rotations in yaw can produce retinal slip when vestibular signals are unreliable^43^, exacerbating oscillopsia. Increased yaw variability may therefore reflect instability in heading regulation under conditions of elevated vestibular prediction error. In this framework, cross-axis kinematic variability does not imply dysfunction of additional canal planes per se, but instead reflects the distributed nature of postural control when one component of the vestibular system becomes unreliable. This interpretation is further supported by the oblique orientation of the semicircular canals, whereby each canal contributes to encoding multiple components of head rotation.

While we observed association between contra-lesional saccular response (cVEMP) and variability of waist vertical acceleration, the above-mentioned systems-level interpretation may also explain why IMU-derived variability showed stronger associations with symptom burden and functional performance than did physiological measures of canal function. Clinical metrics such as vHIT gain or dynamic visual acuity probe specific reflex pathways under controlled conditions. By contrast, kinematic variability during quiet stance reflects how altered vestibular reliability is integrated with visual and somatosensory inputs to stabilize the head and trunk during the multidimensional movements involved in natural standing and balance tasks. Prior studies in vestibular hypofunction have similarly reported inconsistent relationships between reflex measures and functional balance performance^19,44–46^. IMU-based kinematic analysis should therefore be viewed as complementary to clinical vestibular testing, providing insight into how vestibular deficits are expressed in whole-body balance strategies rather than isolated reflex pathways.

If IMU-derived amplitude and variability reflect integrative postural control under altered vestibular reliability, then between-group differences should be most pronounced under conditions that maximize dependence on vestibular input. Consistent with this prediction, the largest and most systematic differences emerged during foam stance with eyes closed, a condition in which visual input is eliminated and somatosensory cues are degraded, increasing reliance on vestibular signals for stabilization (reviewed in^2,47,48^). Visual input normally reduces sway variability and stabilizes posture, although its stabilizing influence declines as sway velocity increases and visual feedback becomes less reliable^49–52^. In individuals with SCDS, poorer visual acuity may further reduce the reliability of visual feedback available for postural stabilization, increasing reliance on vestibular and somatosensory cues. When vestibular input is also altered by the dehiscent canal, this sensory context may contribute to greater variability of head and waist motion.

This sensory context also provides a framework for understanding the multiplanar kinematic variability observed in individuals with SCDS. Vestibular signals contribute to postural stabilization through vestibulospinal pathways, and compensation following vestibular loss involves increased weighting of extra-vestibular inputs within vestibular nuclei circuits (reviewed in^53,54^). When visual and somatosensory cues are simultaneously unreliable, these compensatory mechanisms may be insufficient, leading to the increased kinematic variability observed here. Notably, a composite kinematic score derived from foam stance with eyes closed, using either the head or waist IMU alone, differentiated individuals with SCDS from healthy controls, highlighting the potential utility of this sensory condition for detecting balance instability associated with canal dysfunction.

Together, these findings help explain why conventional vestibular and functional balance assessments may benefit from being complemented by multidimensional kinematic measures in individuals with SCDS. Performance duration differed significantly between groups only during tandem stance with eyes closed, whereas 6DoF kinematic measures revealed differences across multiple balance tasks. Thus, gross task completion can appear relatively preserved even when the structure of postural control is altered. Prior work has similarly shown that quantitative posturographic measures can provide information beyond conventional clinical assessments^55,56^. Our findings extend this idea to SCDS, showing that wearable sensor-based kinematics provide a more detailed characterization of balance impairment than time-based task performance alone.

We further examined relationships between kinematic and clinical measures. One of the strongest and most consistent correlations was observed between the standard deviation of head yaw velocity and measures of oscillopsia (OFI) and persistent postural-perceptual dizziness (PPPD), linking greater head yaw instability during stance with more severe perceptual symptoms. In contrast, IMU-derived kinematic variability showed little relationship with VOR gain or dynamic visual acuity, suggesting that whole-body balance variability reflects integrative control processes rather than isolated semicircular canal physiology. Among physiological measures, greater contra-lesional cVEMP amplitude was associated with greater variability of waist vertical acceleration and yaw velocity, suggesting that otolith-related measures may relate to postural kinematic variability in SCDS, although this exploratory relationship should be interpreted cautiously. Among functional measures, the strongest association was between the standard deviation of waist yaw velocity and Functional Gait Assessment (FGA) score. This finding is consistent with prior work showing that body-segment kinematics, including trunk roll and pitch velocities, can distinguish balance impairment in vestibular populations^57^, and extends this framework to unilateral SCDS.

### Limitations and future directions

By combining head and waist kinematics with clinical and functional measures, the present study provides a detailed multidimensional characterization of standing balance in individuals with unilateral SCDS. This approach revealed altered postural kinematics across multiple axes and sensory conditions, while also identifying several directions for future work. First, because this was a single-center study with a modest sample size and no a priori power analysis, future studies in larger, multi-center cohorts will be important for establishing the generalizability of these findings. Second, although all participants in the SCDS group had unilateral symptoms, some had bilateral bony defects, and all were surgical candidates who had not yet undergone repair at the time of assessment. Because the study was designed to compare unilateral SCDS with healthy controls rather than to test differences between anatomical subgroups, subgroup comparisons of clinical characteristics between participants with unilateral versus bilateral bony defects should be interpreted as exploratory. Extending this approach to individuals with bilateral SCDS and to patients assessed before and after surgical repair will help determine how multiplanar kinematic instability relates to symptom laterality, surgical intervention, and recovery. Finally, examining balance during more natural, real-world behaviors will help define how the kinematic signatures identified here relate to everyday functional impairment and rehabilitation outcomes.

In summary, our findings demonstrate that unilateral SCDS alters head and postural kinematics during quiet standing, particularly when visual and somatosensory feedback are reduced. Compared with conventional functional balance assessments alone, wearable kinematic analysis provides a more detailed and multiplanar characterization of balance impairments, linking altered vestibular reliability to functional disability during standing behavior. This approach may help identify specific movement features that contribute to imbalance in SCDS and inform future studies aimed at improving clinical assessment, rehabilitation, and fall prevention strategies.

## Supplementary Information

Supplementary Information.

## Data Availability

The raw data used in the present study is not publicly available, as the participants in this research did not consent to share their individual data publicly. However, the data may be available from the corresponding author upon reasonable request and approval from the Institutional Review Board.

## References

[1] Horak, F. B. Postural orientation and equilibrium: What do we need to know about neural control of balance to prevent falls?. *Age Ageing* **35**(suppl_2), ii7–ii11. 10.1093/ageing/afl077 (2006). 16926210

[2] Peterka, R. J. Sensorimotor integration in human postural control. *J. Neurophysiol.* **88**(3), 1097–1118. 10.1152/jn.2002.88.3.1097 (2002). 12205132

[3] Cullen, K. E. Vestibular motor control. In *Handbook of Clinical Neurology* Vol. 195 (ed. Younger, D. S.) 31–54 (Elsevier, 2023). 10.1016/B978-0-323-98818-6.00022-4. 37562876

[4] Goldberg, J. M. & Cullen, K. E. Vestibular control of the head: Possible functions of the vestibulocollic reflex. *Exp. Brain Res.* **210**, 331–345. 10.1007/s00221-011-2611-5 (2011). PMC4157641 21442224

[5] Keshner, E. A. & Cohen, H. Current concepts of the vestibular system reviewed: 1. The role of the vestibulospinal system in postural control. *Am. J. Occup. Ther.* **43**(5), 320–330. 10.5014/ajot.43.5.320 (1989). 2655458

[6] Peusner, K. D., Shao, M., Reddaway, R. & Hirsch, J. C. Basic concepts in understanding recovery of function in vestibular reflex networks during vestibular compensation. *Front. Neurol.* **3**. 10.3389/fneur.2012.00017 (2012). PMC3282297 22363316

[7] Agrawal, Y., Carey, J. P., Della Santina, C. C., Schubert, M. C. & Minor, L. B. Disorders of balance and vestibular function in US adults: Data from the National Health and Nutrition Examination Survey, 2001-2004. *Arch. Intern. Med.* **169**(10), Article 10. 10.1001/archinternmed.2009.66 (2009). 19468085

[8] Donovan, J., De Silva, L., Cox, H., Palmer, G. & Semciw, A. I. Vestibular dysfunction in people who fall: A systematic review and meta-analysis of prevalence and associated factors. *Clin. Rehabil.* **37**(9), 1229–1247. 10.1177/02692155231162423 (2023). 37036433

[9] Minor, L. B. Superior canal dehiscence syndrome. *Am.manifestations of superior semicircular J. Otol.* **21**(1), 9–19 (2000). 10651428

[10] Minor, L. B. Clinical manifestations of superior semicircular canal dehiscence. *Laryngoscope* **115**(10), 1717–1727. 10.1097/01.mlg.0000178324.55729.b7 (2005). 16222184

[11] Ward, B. K. et al. Superior semicircular canal dehiscence syndrome: Diagnostic criteria consensus document of the committee for the classification of vestibular disorders of the Bárány Society. *J. Vestib. Res.* **31**(3), 131–141. 10.3233/VES-200004 (2021). PMC9249274 33522990

[12] Ocak, I. et al. Impact of superior canal dehiscence syndrome on health utility values: A prospective case-control study. *Front. Neurol.* **11**, 552495. 10.3389/fneur.2020.552495 (2020). PMC7578361 33133004

[13] Öhman, J., Forssén, A., Sörlin, A. & Tano, K. Patients’ experiences of living with superior canal dehiscence syndrome. *Int. J. Audiol.* **57**(11), 825–830. 10.1080/14992027.2018.1487086 (2018). 30178689

[14] Janky, K. L., Zuniga, M. G., Carey, J. P. & Schubert, M. Balance dysfunction and recovery after surgery for superior canal dehiscence syndrome. *Arch. Otolaryngol. Head Neck Surg.* **138**(8), 723–730. 10.1001/archoto.2012.1329 (2012). PMC3632400 22801722

[15] Aryan, R. et al. Superior canal dehiscence syndrome induces canal-specific kinematic adaptations during locomotion. *Sci. Rep.* **15**(1), 33356. 10.1038/s41598-025-16904-7 (2025). PMC12480983 41022820

[16] Zobeiri, O. A., Mischler, G. M., King, S. A., Lewis, R. F. & Cullen, K. E. Effects of vestibular neurectomy and neural compensation on head movements in patients undergoing vestibular schwannoma resection. *Sci. Rep.* **11**(1), 517. 10.1038/s41598-020-79756-3 (2021). PMC7804855 33436776

[17] Halmagyi, G. M. et al. The video head impulse test. *Front. Neurol.* **8**, 258. 10.3389/fneur.2017.00258 (2017). PMC5465266 28649224

[18] Nguyen, K. D., Welgampola, M. S. & Carey, J. P. Test-retest reliability and age-related characteristics of the ocular and cervical vestibular evoked myogenic potential tests. *Otol. Neurotol.* **31**(5), 793–802. 10.1097/MAO.0b013e3181e3d60e (2010). PMC2913294 20517167

[19] Millar, J. L., Gimmon, Y., Roberts, D. & Schubert, M. C. Improvement after vestibular rehabilitation not explained by improved passive VOR gain. *Front. Neurol.* **11**, 79. 10.3389/fneur.2020.00079 (2020). PMC7044341 32153490

[20] Rine, R. M. et al. Vestibular function assessment using the NIH toolbox. *Neurology* **80**(11 Suppl 3), S25–S31. 10.1212/WNL.0b013e3182872c6a (2013). PMC3662339 23479540

[21] Whitney, S. L., Marchetti, G. F., Schade, A. & Wrisley, D. M. The sensitivity and specificity of the Timed “Up & Go” and the Dynamic Gait Index for self-reported falls in persons with vestibular disorders. *J. Vestib. Res.: Equilib. Orientat.* **14**(5), 397–409 (2004). 15598995

[22] Wrisley, D. M., Marchetti, G. F., Kuharsky, D. K. & Whitney, S. L. Reliability, internal consistency, and validity of data obtained with the functional gait assessment. *Phys. Ther.* **84**(10), 906–918. 10.1093/ptj/84.10.906 (2004). 15449976

[23] Powell, L. E. & Myers, A. M. The activities-specific balance confidence (ABC) scale. *J. Gerontol.: Ser. A* **50A**(1), 1. 10.1093/gerona/50A.1.M28 (1995). 7814786

[24] Beck, A. T., Epstein, N., Brown, G. & Steer, R. A. An inventory for measuring clinical anxiety: Psychometric properties. *J. Consult. Clin. Psychol.* **56**(6), 6. 10.1037/0022-006X.56.6.893 (1988). 3204199

[25] Kosinski, M. et al. A six-item short-form survey for measuring headache impact: The HIT-6. *Qual. Life Res.: Int. J. Qual. Life Asp. Treat., Care Rehabilit.* **12**(8), 963–974. 10.1023/a:1026119331193 (2003). 14651415

[26] Anson, E. R., Gimmon, Y., Kiemel, T., Jeka, J. J. & Carey, J. P. A tool to quantify the functional impact of oscillopsia. *Front. Neurol.* **9**, 142. 10.3389/fneur.2018.00142 (2018). PMC5862789 29599743

[27] Yagi, C. et al. A validated questionnaire to assess the severity of persistent postural-perceptual dizziness (PPPD): The Niigata PPPD questionnaire (NPQ). *Otol. Neurotol.* **40**(7), e747–e752. 10.1097/MAO.0000000000002325 (2019). PMC6641087 31219964

[28] Jacobson, G. P. & Newman, C. W. The development of the Dizziness Handicap Inventory. *Arch. Otolaryngol. -Head Neck Surg.* **116**(4), 4. 10.1001/archotol.1990.01870040046011 (1990). 2317323

[29] Unseld, T., Ruckerbauer, L. & Mayer, B. Permutation tests are a useful alternative approach for statistical hypothesis testing in small sample sizes. *Altern. Lab. Anim.* **53**(3), 130–137. 10.1177/02611929251326882 (2025). 40118101

[30] Ward, B. K., Carey, J. P. & Minor, L. B. Superior canal dehiscence syndrome: Lessons from the first 20 years. *Front. Neurol.* **8**, 177. 10.3389/fneur.2017.00177 (2017). PMC5408023 28503164

[31] Horak, F. B. Clinical assessment of balance disorders. *Gait Posture* **6**(1), 76–84. 10.1016/S0966-6362(97)00018-0 (1997).

[32] Horak, F. B., Henry, S. M. & Shumway-Cook, A. Postural perturbations: New insights for treatment of balance disorders. *Phys. Ther.* **77**(5), 517–533. 10.1093/ptj/77.5.517 (1997). 9149762

[33] Mergner, T. & Rosemeier, T. Interaction of vestibular, somatosensory and visual signals for postural control and motion perception under terrestrial and microgravity conditions—a conceptual model. *Brain Res. Brain Res. Rev.* **28**(1–2), 118–135. 10.1016/s0165-0173(98)00032-0 (1998). 9795180

[34] Winter, D. A., Prince, F., Frank, J. S., Powell, C. & Zabjek, K. F. Unified theory regarding A/P and M/L balance in quiet stance. *J. Neurophysiol.* **75**(6), 2334–2343. 10.1152/jn.1996.75.6.2334 (1996). 8793746

[35] Winter, D., Prince, F., Stergiou, P. & Powell, C. Medial-lateral and anterior-posterior motor responses associated with centre of pressure changes in quiet standing. *Neurosci. Res. Commun.* **12**(3), 3 (1993).

[36] Horak, F. B. & Macpherson, J. M. Postural orientation and equilibrium. In *Comprehensive Physiology* 255–292 (Wiley, 1995). 10.1002/j.2040-4603.1995.tb01704.x.

[37] Day, B. L., Séverac Cauquil, A., Bartolomei, L., Pastor, M. A. & Lyon, I. N. Human body-segment tilts induced by galvanic stimulation: A vestibularly driven balance protection mechanism. *J. Physiol.* **500**, 661–672. 10.1113/jphysiol.1997.sp022051 (1997). PMC1159417 9161984

[38] Fitzpatrick, R. C. & Day, B. L. Probing the human vestibular system with galvanic stimulation. *J. Appl. Physiol.* **96**(6), 2301–2316. 10.1152/japplphysiol.00008.2004 (2004). 15133017

[39] Forbes, P. A., Chen, A. & Blouin, J.-S. Sensorimotor control of standing balance. In *Handbook of Clinical Neurology * Vol. 159, 61–83 (Elsevier, 2018). 10.1016/B978-0-444-63916-5.00004-5. 30482333

[40] Karmali, F. et al. The role of vestibular cues in postural sway. *J. Neurophysiol.* **125**(2), 672–686. 10.1152/jn.00168.2020 (2021). PMC7948142 33502934

[41] Angelaki, D. E. & Cullen, K. E. Vestibular system: The many facets of a multimodal sense. *Annu. Rev. Neurosci.* **31**, 125–150. 10.1146/annurev.neuro.31.060407.125555 (2008). 18338968

[42] Goldberg, J. M. et al. *The Vestibular System: A Sixth Sense* (Oxford University Press, 2012).

[43] Leigh, J. R. & Zee, D. S. *The Neurology of Eye Movements* 5th edition. (Oxford University Press, 2015).

[44] Evans, M. K. & Krebs, D. E. Posturography does not test vestibulospinal function. *Otolaryngol. Head Neck Surg.* **120**(2), 164–173. 10.1016/S0194-5998(99)70401-8 (1999). 9949347

[45] Sprenger, A., Wojak, J. F., Jandl, N. M. & Helmchen, C. Postural control in bilateral vestibular failure: Its relation to visual, proprioceptive, vestibular, and cognitive input. *Front. Neurol.* **8**, 444. 10.3389/fneur.2017.00444 (2017). PMC5585141 28919878

[46] Zobeiri, O. A., Wang, L., Millar, J. L., Schubert, M. C. & Cullen, K. E. Head movement kinematics are altered during balance stability exercises in individuals with vestibular schwannoma. *J. Neuroeng. Rehabil.* **19**(1), 120. 10.1186/s12984-022-01109-0 (2022). PMC9648040 36352393

[47] Halmágyi, G. M. & Curthoys, I. S. Vestibular contributions to the Romberg test: Testing semicircular canal and otolith function. *Eur. J. Neurol.* **28**(9), 3211–3219. 10.1111/ene.14942 (2021). 34160115

[48] Horak, F. B. Postural compensation for vestibular loss. *Ann. N. Y. Acad. Sci.* **1164**(1), 76–81. 10.1111/j.1749-6632.2008.03708.x (2009). PMC3224857 19645883

[49] Assländer, L., Hettich, G. & Mergner, T. Visual contribution to human standing balance during support surface tilts. *Hum. Mov. Sci.* **41**, 147–164. 10.1016/j.humov.2015.02.010 (2015). PMC4427279 25816794

[50] Assländer, L. & Peterka, R. J. Sensory reweighting dynamics in human postural control. *J. Neurophysiol.* **111**(9), 1852–1864. 10.1152/jn.00669.2013 (2014). PMC4044370 24501263

[51] Assländer, L. & Peterka, R. J. Sensory reweighting dynamics following removal and addition of visual and proprioceptive cues. *J. Neurophysiol.* **116**(2), 272–285. 10.1152/jn.01145.2015 (2016). PMC4969387 27075544

[52] Rasman, B. G., Forbes, P. A., Tisserand, R. & Blouin, J.-S. Sensorimotor manipulations of the balance control loop–beyond imposed external perturbations. *Front. Neurol.* **9**, 899. 10.3389/fneur.2018.00899 (2018). PMC6212554 30416481

[53] Cullen, K. E. Vestibular processing during natural self-motion: Implications for perception and action. *Nat. Rev. Neurosci.* **20**, 346–363. 10.1038/s41583-019-0153-1 (2019). PMC6611162 30914780

[54] Sadeghi, S. G., Minor, L. B. & Cullen, K. E. Multimodal integration after unilateral labyrinthine lesion: Single vestibular nuclei neuron responses and implications for postural compensation. *J. Neurophysiol.* **105**(2), 661–673. 10.1152/jn.00788.2010 (2011). PMC3059170 21148096

[55] Mancini, M. & Horak, F. B. The relevance of clinical balance assessment tools to differentiate balance deficits. *Eur. J. Phys. Rehabil. Med.* **46**(2), 239–248 (2010). PMC3033730 20485226

[56] Whitney, S. L., Marchetti, G. F. & Schade, A. I. The relationship between falls history and computerized dynamic posturography in persons with balance and vestibular disorders. *Arch. Phys. Med. Rehabil.* **87**(3), 402–407. 10.1016/j.apmr.2005.11.002 (2006). 16500176

[57] Allum, J. H. et al. Trunk sway measures of postural stability during clinical balance tests: Effects of a unilateral vestibular deficit. *Gait Posture* **14**(3), 227–237. 10.1016/s0966-6362(01)00132-1 (2001). 11600326

