# Supplementary material for: Superior canal dehiscence syndrome alters kinematics of head and postural control during standing
Source: Sci Rep. 2026 Aug 8;16:37491. doi: 10.1038/s41598-026-63082-1 (PMC13747815; doi:10.1038/s41598-026-63082-1)
Supplement: Supplementary file 1 [file 41598_2026_63082_MOESM1_ESM.pdf]

Supplementary Materials

Title: Superior canal dehiscence syndrome alters kinematics of head and postural control during standing

Authors: Raabeae Aryan[1], Jennifer L. Millar[2], John P. Carey[3], Michael C. Schubert[2,3], Kathleen E. Cullen[1,3,4,5,*]

Affiliations:

1) Department of Biomedical Engineering, Johns Hopkins University School of Medicine, Baltimore, MD, United States; 2) Department of Physical Medicine and Rehabilitation, Johns Hopkins University School of Medicine, Baltimore, MD, United States; 3) Department of Otolaryngology-Head and Neck Surgery, Johns Hopkins University School of Medicine, Baltimore, MD, United States; 4) Department of Neuroscience, Johns Hopkins University School of Medicine, Baltimore, MD, United States; 5) Kavli Neuroscience Discovery Institute, Johns Hopkins University, Baltimore, MD, United States.

***Corresponding Author:** Dr. Kathleen E. Cullen; Email: Kathleen.Cullen@jhu.edu

Conflict of Interests: None

Supplementary Figure 1

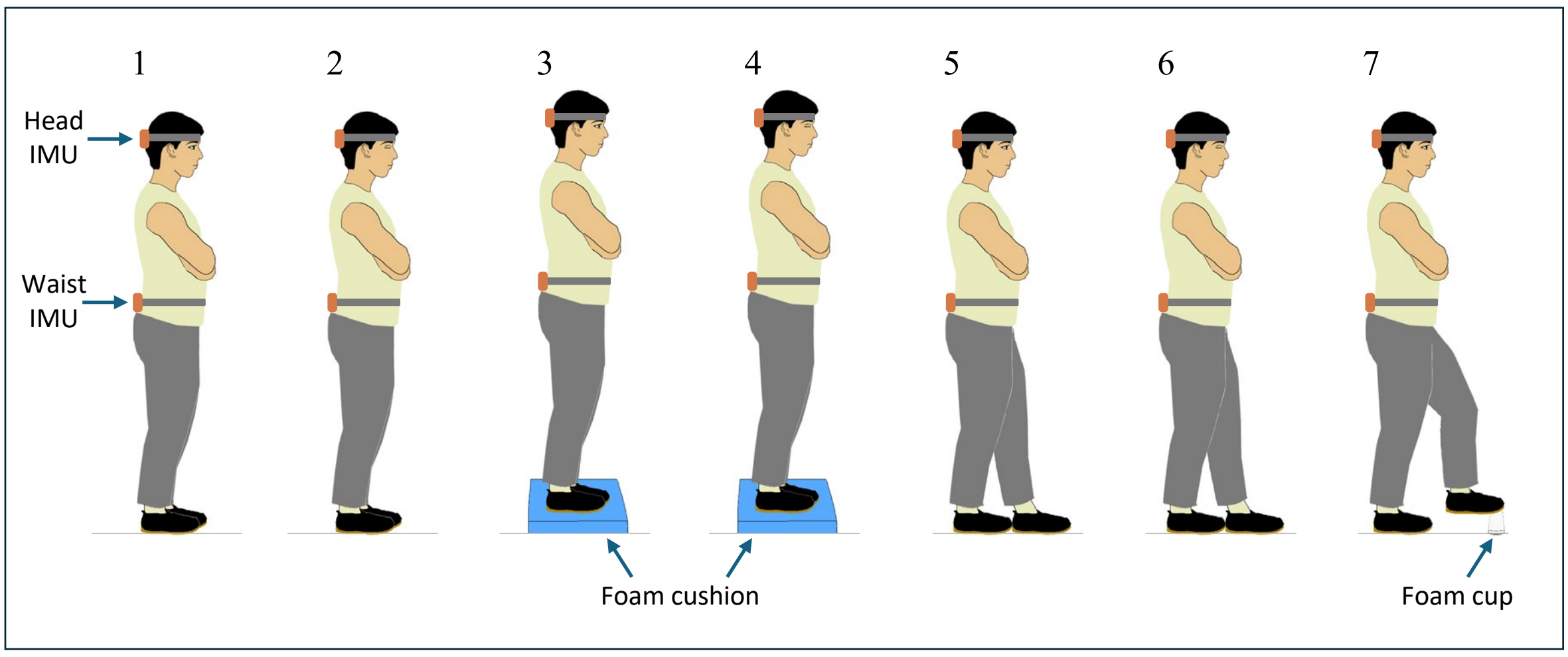

Supplementary Figure 1: Schematic representation of seven balance tasks performed in this study: **1)** Standing on firm surface with eyes open; **2)** Standing on firm surface with eyes closed; **3)** Standing on foam cushion with eyes open; **4)** Standing on foam cushion with eyes closed; **5)** Tandem stance with eyes open; **6)** Tandem stance with eyes closed; and **7)** Single-leg foam-cup stance. Each of the balance tasks were performed for maximum 30 seconds. In task condition 7, participants stood with one foot on the ground and the other on top of a foam cup (without crushing the cup) for 15 seconds, they then switched feet and continued for an additional 15 seconds.

Supplementary Figure 2

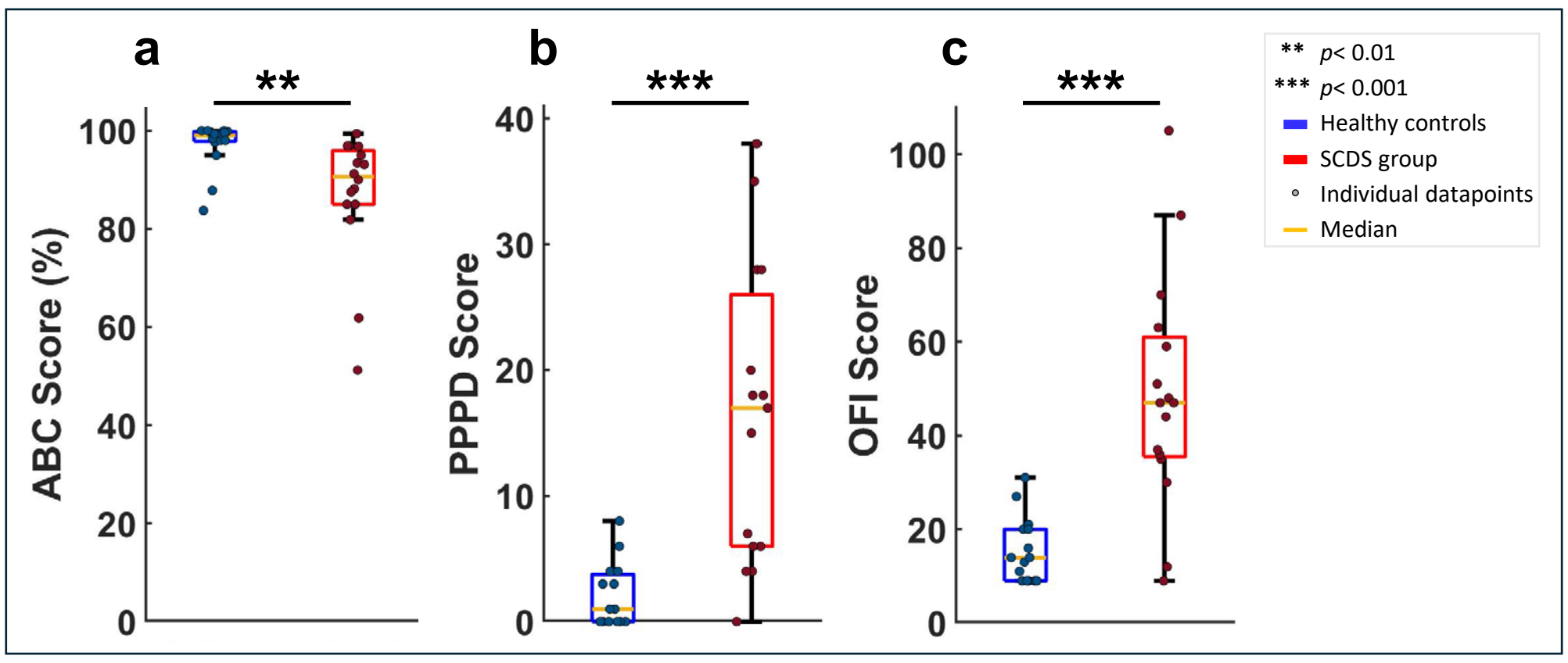

Supplementary Figure 2: Comparison of participant-perceived outcomes measures between the unilateral SCDS (red boxes and dots) and healthy control groups (blue boxes and dots): **a)** ABC (Activities-specific Balance Confidence questionnaire), **b)** PPPD (Niigata Persistent Postural-Perceptual Dizziness questionnaire), and **c)** OFI (Oscillopsia Functional Impact questionnaire). The asterisks represent the significance of between-group differences on permutation test comparison, orange lines represent the median of each group, and the scatter dots represent the individual datapoints for each group. ** *p-value*< 0.01, and *** *p-value*< 0.001.

Supplementary Figure 3

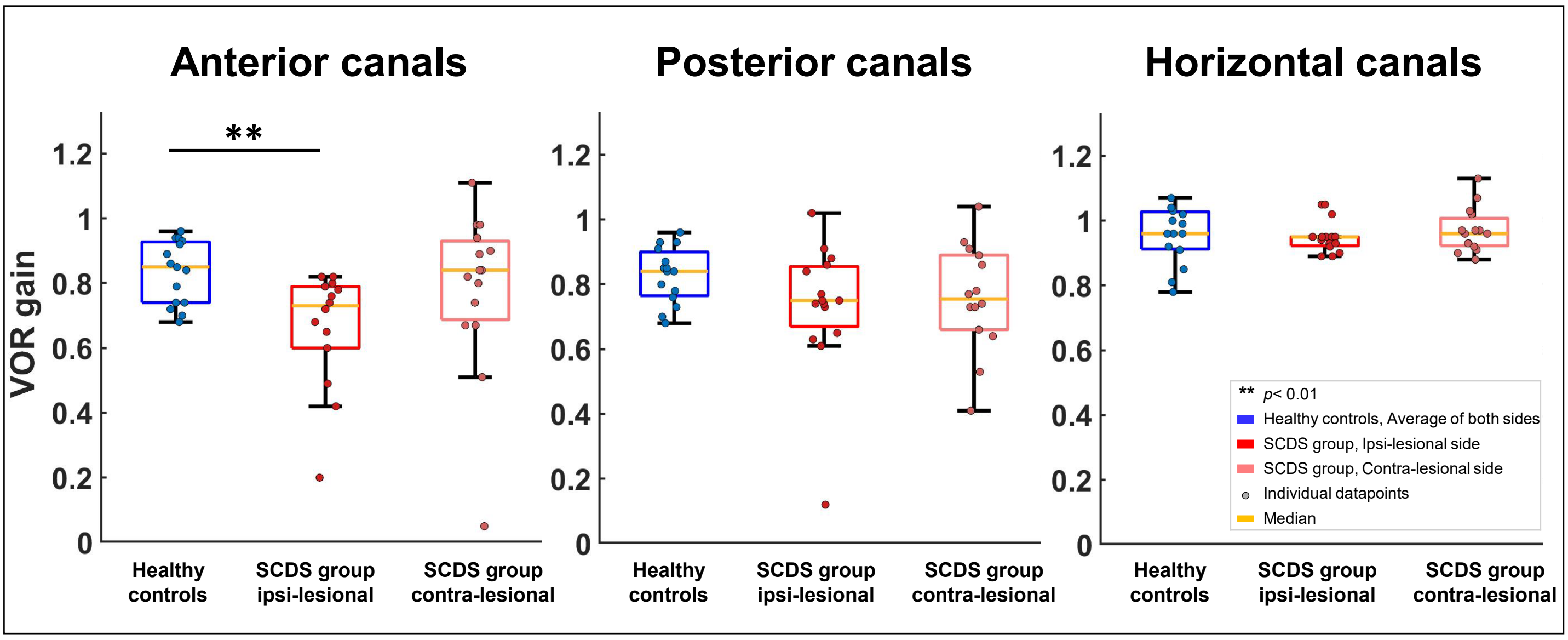

Supplementary Figure 3: Comparison analysis of vHIT-induced passive VOR gains extracted from the "anterior", "posterior", and "horizontal" semicircular canals of the healthy controls (blue boxes and dots) against the SCDS group's ipsi-lesional (red boxes and dots), and contra-lesional (pink boxes and dots) canals. The values presented for healthy individuals are the average of their right and left sides for corresponding canals. The asterisks represent the significance of differences on permutation test comparison, the orange lines represent the median of each group, and the scatter dots represent the individual datapoints for each group. ** p-value< 0.01.

Supplementary Figure 4

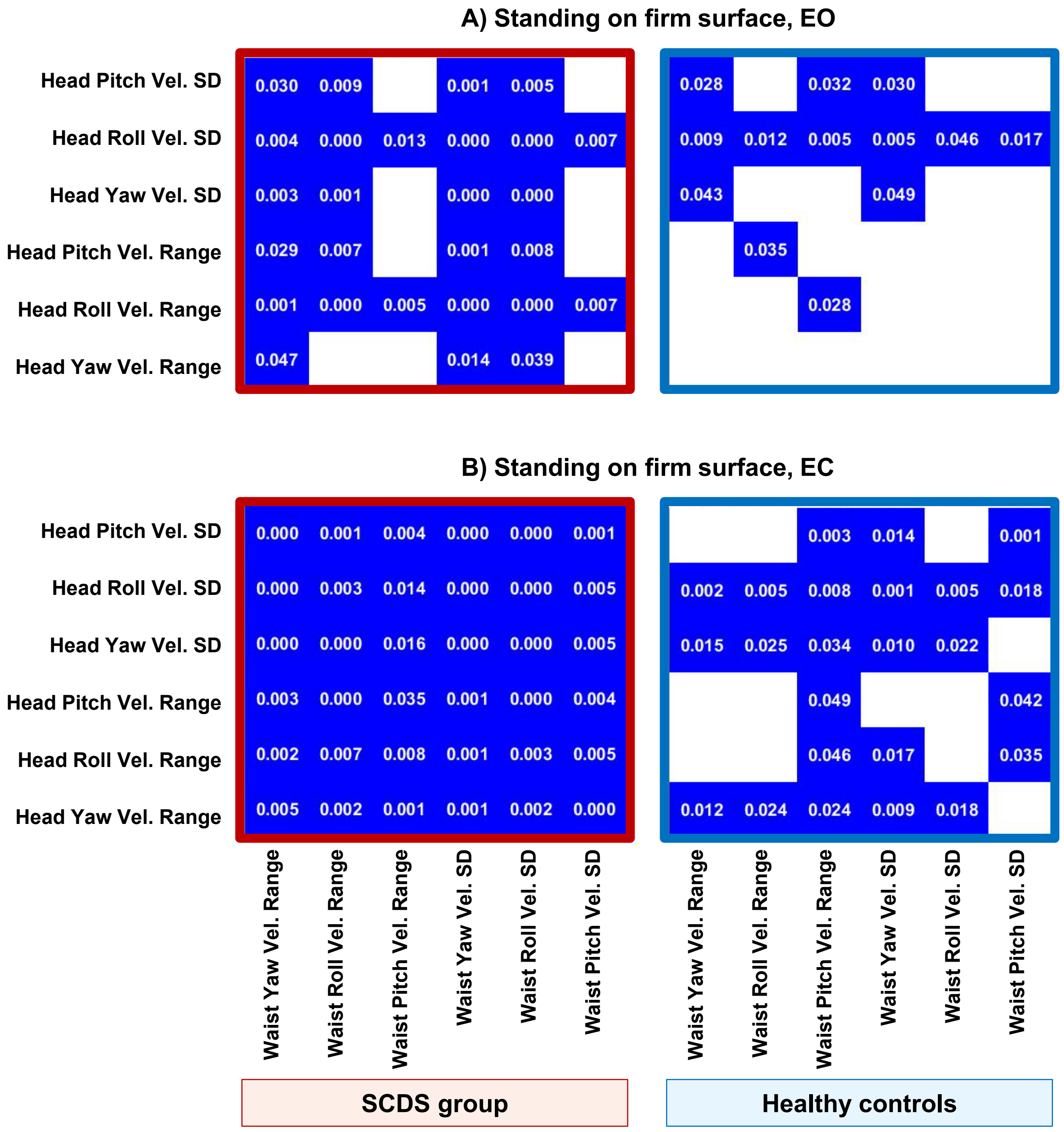

Supplementary Figure 4: Values presented in each cell are the p-values of correlational analysis, and blue cells indicate positive correlations. SCDS: superior canal dehiscence syndrome. EO: eyes open. EC: eyes closed. Vel: velocity. SD: standard deviation. α= 0.05.

Supplementary Figure 5

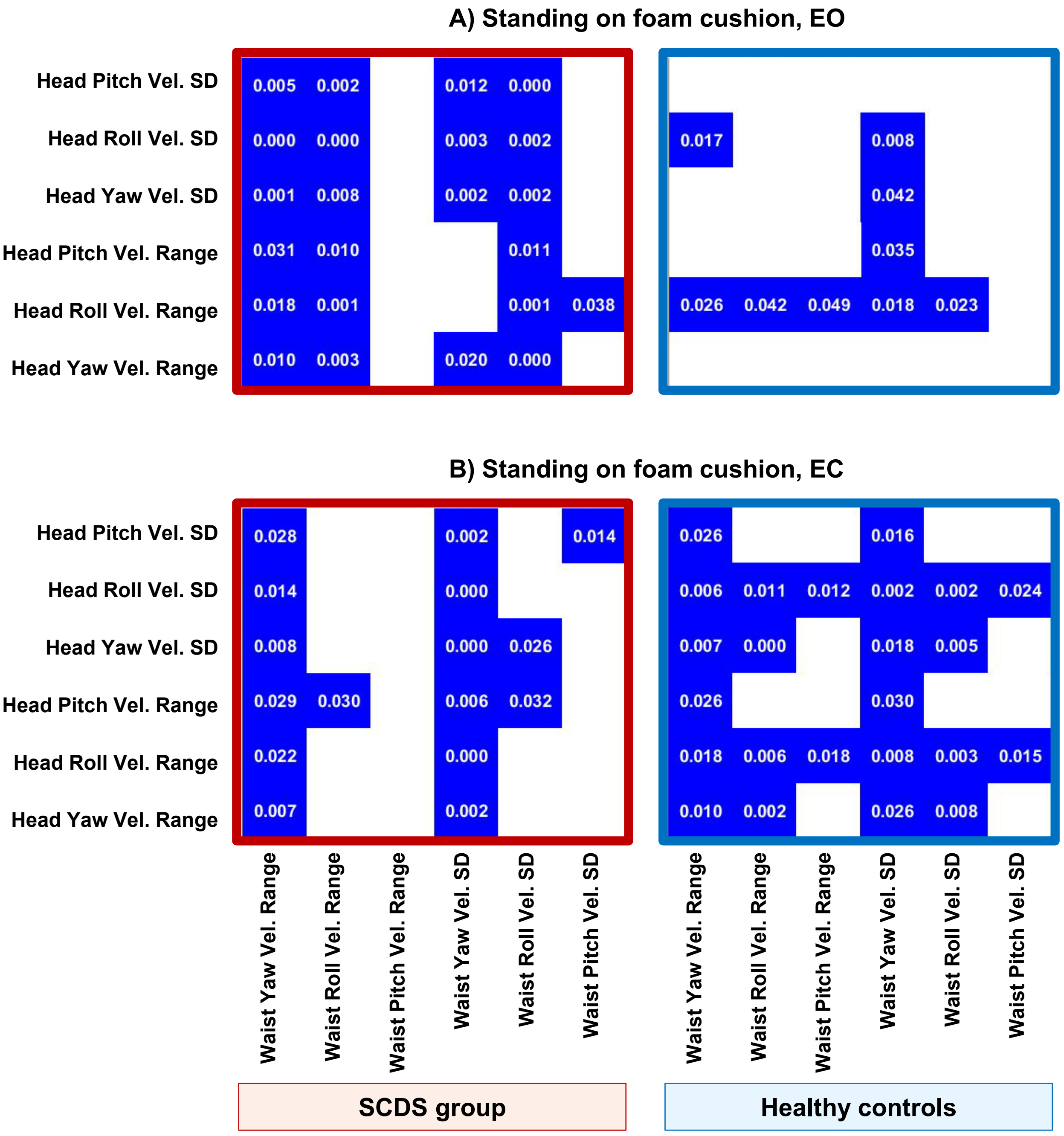

Supplementary Figure 5: Values presented in each cell are the p-values of correlational analysis, and blue cells indicate positive correlations. SCDS: superior canal dehiscence syndrome. EO: eyes open. EC: eyes closed. Vel: velocity. SD: standard deviation. α= 0.05.

Supplementary Figure 6

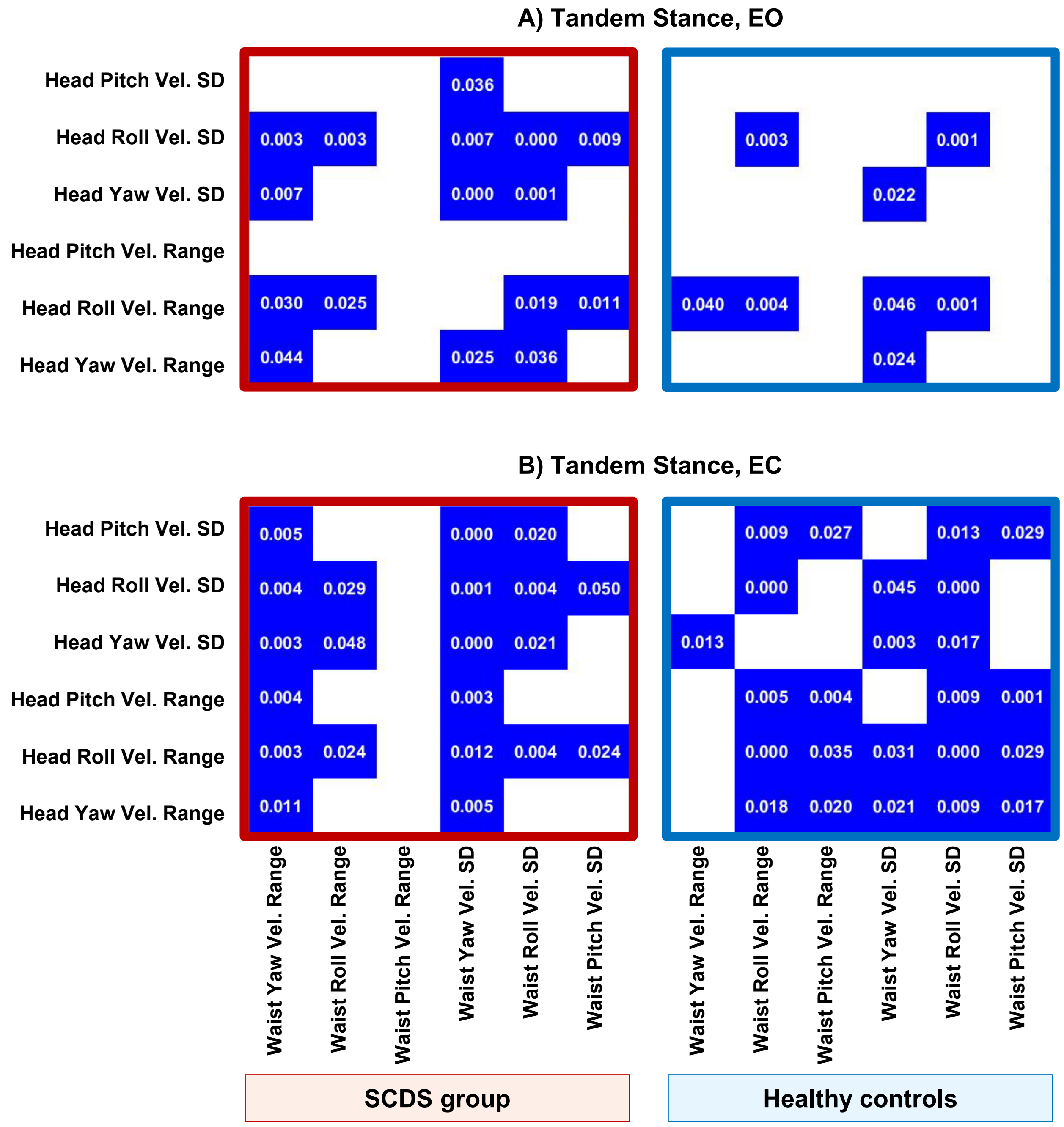

Supplementary Figure 6: Values presented in each cell are the p-values of correlational analysis, and blue cells indicate positive correlations. SCDS: superior canal dehiscence syndrome. EO: eyes open. EC: eyes closed. Vel: velocity. SD: standard deviation. α= 0.05.

Supplementary Figure 7

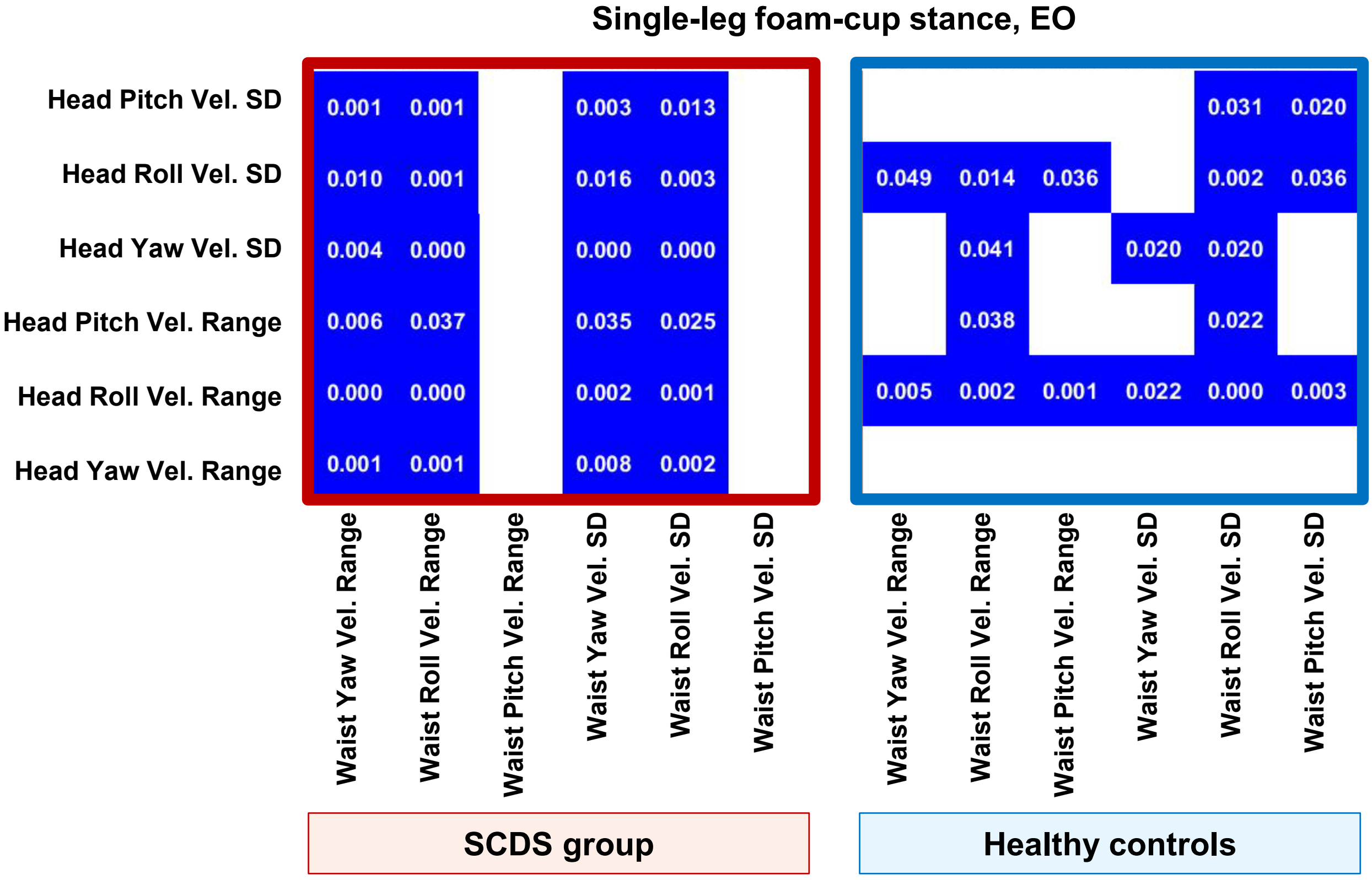

Supplementary Figure 7: Values presented in each cell are the p-values of correlational analysis, and blue cells indicate positive correlations. SCDS: superior canal dehiscence syndrome. EO: eyes open. Vel: velocity. SD: standard deviation. α= 0.05.

Supplementary Table 1: Participants' demographic and clinical characteristics.

Clinical Measures	[a] n SCDS, n Controls	SCDS Group	Healthy Controls	p
**Age** (years)	16,16	46.8 ± 11.2	47.3 ± 11.0	*ns*
**Sex** (M, F)		6, 10	6, 10	-
**Height** (cm)	16,15	169.8 ± 9.7	168.4 ± 10.1	*ns*
**Weight** (lbs.)	16,15	188.6 ± 40.9	168.9 ± 39.2	*ns*
**Symptomatic side** (R, L)		6, 10	na	-
**FGA** (score)	16,16	25.19 ± 3.66	28.81 ± 1.17	***
**TUG** (s)	16,16	10.15 ± 1.28	8.00 ± 1.26	***
**TUG.Cog** (s)	14,12	12.98 ± 3.68	9.58 ± 1.70	**
**Gait speed** (m/s)	16,16	1.22 ± 0.17	1.41 ± 0.16	**
**ABC** (%)	16,16	87.09 ± 13.06	97.20 ± 4.70	**
**OFI** (score)	16,15	48.75 ± 24.76	15.47 ± 7.00	***
**PPPD** (score)	15,15	16.27 ± 11.90	2.00 ± 2.56	***
**DHI** (score)	16,16	31.50 ± 19.86	2.00 ± 4.56	***
**HIT-6** (score)	16,16	48.88 ± 8.86	43.50 ± 8.63	*ns*
**BAI** (score)	16,16	13.88 ± 9.91	4.38 ± 7.92	**
**Autophony** (score)	8,7	32.13 ± 27.55	0	***
**LogMAR** (score)				
Static	16,16	-0.14 ± 0.09	-0.11 ± 0.11	*ns*
Ipsi-Horizontal‡	16,16	0.27 ± 0.13	0.21 ± 0.08 †	*ns*
Contra-Horizontal‡	16,16	0.31 ± 0.15		*
Upward‡	15,15	0.26 ± 0.14	0.20 ± 0.12	*ns*
Downward‡	15,14	0.31 ± 0.13	0.19 ± 0.12	*

Presented values are means± standard deviations for continuous variables and counts for categorical variables. [a] This column represents each group's available data (out of 16) for the corresponding variable. **FGA:** Functional Gait Assessment. **TUG:** Timed Up and Go (the value presented is the average of one trial with ipsi-lesional turn and one trial with contra-lesional turn). **TUG.Cog:** Timed Up and Go while counting backwards by three (the value presented is the average of one trial with ipsi-lesional turn and one trial with contra-lesional turn). **ABC:** Activities-specific Balance Confidence questionnaire. **OFI:** Oscillopsia Functional Impact. **PPPD:** Niigata Persistent Postural-Perceptual Dizziness questionnaire. **DHI:** Dizziness Handicap Inventory. **HIT-6:** Headache Impact Test. **BAI:** Beck Anxiety Inventory. **Ipsi:** Denotes the symptomatic side for SCDS group. **Contra:** Denotes the asymptomatic side for SCDS group. **LogMAR:** (Logarithm of the minimum angle of resolution) Logarithmic visual acuity score based on the dynamic visual acuity (DVA) test. **‡:** Values presented are corrected LogMar scores. **†:** Horizontal LogMar score presented for the healthy control group is the average of corrected horizontal Logmar scores of their right and left sides. **na:** not applicable. Significance level α=0.05. **ns:** not significant. *p-value<0.05, **p-value<0.01, ***p-value<0.001. ‡Corrected LogMAR scores.

Supplementary Tables 2: Participants' vestibular physiological measures.

Clinical Measures	[a] n SCDS, n Controls	SCDS Group	Control Group [c]	p
**vHIT VOR Gain**				
Ipsi-Horizontal	15, 15	0.95 ± 0.05	0.96 ± 0.09	*ns*
Contra-Horizontal	15, 15	0.97 ± 0.07		*ns*
Ipsi-Posterior	15, 15	0.73 ± 0.20	0.83 ± 0.09	*ns*
Contra-Posterior	14, 15	0.76 ± 0.17		*ns*
Ipsi-Anterior	14, 15	0.66 ± 0.18	0.83 ± 0.10	**
Contra-Anterior	15, 15	0.78 ± 0.25		*ns*
**VEMPs** [b]				
Ipsi-cVEMP Amp.	15, na	5.10 ± 6.45	-	-
Contra-cVEMP Amp.	15, na	2.66 ± 3.04	-	-
cVEMP Asymmetry (%)	15, na	30.63 ± 21.25	-	-
Ipsi-oVEMP Amp. (µV)	14, na	18.21 ± 14.30	-	-
Contra-oVEMP Amp. (µV)	13, na	5.18 ± 7.84	-	-
oVEMP Asymmetry (%)	13, na	57.72 ± 30.82	-	-

Presented values are means± standard deviations for continuous variables and counts for categorical variables. **a:** This column represents each group's available data (out of 16) for the corresponding variable. **b:** VEMP amplitudes were extracted from the SCDS group's medical records if results were available; however, they were not assessed in the healthy control group. **c:** VOR gain values presented for healthy individuals are the average of their right and left sides for corresponding canals. **vHIT:** video Head Impulse Test. **VOR:** Vestibulo-ocular reflex. **Ipsi:** symptomatic side for SCDS group. **Contra:** asymptomatic side for SCDS group. **VEMPs:** Vestibular Evoked Myogenic Potentials. **cVEMP:** Cervical Vestibular Evoked Myogenic Potential. **oVEMP:** Ocular Vestibular Evoked Myogenic Potential. Presented values are means± standard deviations. **Amp.:** Amplitude; amplitudes for cVEMP are rectified values. **Asymmetry:** Amplitudes were extracted from the SCDS group's medical records; accordingly, VEMP asymmetry ratios for each SCDS participant was calculated as the absolute value of 100*($Amp._{Right}$ – $Amp._{Left}$)/($Amp._{Right}$ + $Amp._{Left}$) for oVEMP; and for cVEMP as the absolute value of 100*(rectified $Amp._{Right}$ – rectified $Amp._{Left}$)/(rectified $Amp._{Right}$ + rectified $Amp._{Left}$). Significance level α=0.05. **na:** not assessed. **ns:** not significant. **p-value<0.01.

Supplementary Table 3: Characteristics of unilateral and bilateral anatomical sub-groups

Clinical Measures	[a] n Unilateral, n Bilateral anatomical SCD	Unilateral anatomical SCD	Bilateral anatomical SCD	p
Age (years)	8, 8	50.62 ± 13.82	42.88 ± 6.58	*ns*
Sex (M, F)		3,5	3,5	-
Height (cm)	8, 8	169.49 ± 9.42	170.20 ± 10.54	*ns*
Weight (lbs.)	8, 8	184.50 ± 38.73	192.62 ± 45.19	*ns*
Symptomatic side (R, L)		4,4	2,6	*na*
FGA (score)	8, 8	25.50 ± 3.42	24.88 ± 4.09	*ns*
TUG (s)	8, 8	9.96 ± 1.06	10.33 ± 1.53	*ns*
TUG.Cog (s)	7, 7	13.06 ± 4.76	12.90 ± 2.57	*ns*
Gait speed (m/s)	8, 8	1.19 ± 0.21	1.24 ± 0.13	*ns*
ABC (%)	8, 8	86.05 ± 14.62	88.13 ± 12.23	*ns*
OFI (score)	8, 8	43.25 ± 19.03	54.25 ± 29.70	*ns*
PPPD (score)	7, 8	16.14 ± 11.80	16.38 ± 12.81	*ns*
DHI (score)	8, 8	28.25 ± 22.21	34.75 ± 18.11	*ns*
HIT-6 (score)	8, 8	50.25 ± 8.68	47.50 ± 9.41	*ns*
BAI (score)	8, 8	15.25 ± 12.87	12.50 ± 6.35	*ns*
Autophony[†] (score)	4, 4	47.75 ± 30.37	16.50 ± 14.06	*na*
LogMAR (score)				
Static	8, 8	-0.17 ± 0.09	-0.11 ± 0.08	*ns*
Ipsi-Horizontal[‡]	8, 8	0.28 ± 0.15	0.26 ± 0.12	*ns*
Contra-Horizontal[‡]	8, 8	0.38 ± 0.19	0.25 ± 0.07	*ns*
Upward[‡]	7, 8	0.28 ± 0.14	0.24 ± 0.15	*ns*
Downward[‡]	7, 8	0.39 ± 0.14	0.25 ± 0.05	*
vHIT VOR Gain				
Ipsi-Horizontal	7, 8	0.95 ± 0.05	0.95 ± 0.06	*ns*
Ipsi-Posterior	7, 8	0.79 ± 0.14	0.69 ± 0.25	*ns*
Ipsi-Anterior	6, 8	0.62 ± 0.13	0.69 ± 0.21	*ns*
Contra-Horizontal	7, 8	0.97 ± 0.08	0.97 ± 0.06	*ns*
Contra-Posterior	6, 8	0.75 ± 0.13	0.77 ± 0.20	*ns*
Contra-Anterior	7, 8	0.83 ± 0.20	0.74 ± 0.30	*ns*
VEMPs				
Ipsi-cVEMP Amp.	7, 8	3.53 ± 4.21	6.48 ± 7.96	*ns*
Contra-cVEMP Amp.	7, 8	1.93 ± 3.02	3.30 ± 3.11	*ns*
cVEMP Asymmetry (%)	7 8	34.70 ± 26.72	27.08 ± 16.11	*ns*
Ipsi-oVEMP Amp. (µV)	6, 8	12.66 ± 11.20	22.37 ± 15.63	*ns*
Contra-oVEMP Amp. (µV)	5, 8	6.75 ± 12.13	4.20 ± 4.30	*ns*
oVEMP Asymmetry (%)	5, 8	51.09 ± 33.52	61.85 ± 30.58	*ns*

Presented values are means± standard deviations for continuous variables, and counts for categorical variables. a: This column represent each sub-group's available data (out of 8) for the corresponding variable. SCD: Superior Canal Dehiscence. FGA: Functional Gait Assessment scale. TUG: Timed Up and Go test. TUG.Cog: Timed Up and Go test while counting backwards by three. ABC: Activities-specific Balance Confidence questionnaire. OFI: Oscillopsia Functional Impact questionnaire. PPPD: Niigata Persistent Postural-Perceptual dizziness questionnaire. DHI: Dizziness Handicap Inventory questionnaire. HIT-6: Headache Impact Test questionnaire. BAI: Beck Anxiety Inventory questionnaire. Ipsi: symptomatic side. Contra: asymptomatic side. LogMAR: Logarithm of the minimum

angle of resolution; Logarithmic visual acuity score based on the Dynamic Visual Acuity (DVA) test. vHIT: video Head Impulse Test. VOR: Vestibulo-ocular reflex. cVEMP: cervical vestibular evoked myogenic potential. oVEMP: ocular vestibular evoked myogenic potential. **Amp.:** Amplitude; amplitudes for cVEMP are rectified values. **Asymmetry:** Amplitudes were extracted from the SCDS group's medical records; accordingly, VEMP asymmetry ratios for each SCDS participant was calculated as the absolute value of 100*($Amp._{Right}$ – $Amp._{Left}$)/($Amp._{Right}$ + $Amp._{Left}$) for oVEMP; and for cVEMP as the absolute value of 100*(rectified $Amp._{Right}$ – rectified $Amp._{Left}$)/(rectified $Amp._{Right}$ + rectified $Amp._{Left}$). †Autophony index data was only available for four participants in each group; therefore, due to the low power a statistical comparison of the autophony index was not performed between sub-groups. ‡Corrected LogMAR score values. Significance level α=0.05. p: p-value. *p< 0.05. na: not assessed. ns: not significant.

Supplementary Table 4: Size of both groups during each balance task.

Balance Tasks	Waist Kinematic Data	Head Kinematic Data [†]	Completion Time Data
	SCDS n; Controls n	SCDS n; Controls n	SCDS n; Controls n
Standing with feet together, Firm surface, EO [a]	16; 12	15; 12	16; 12
Standing with feet together, Firm surface, EC	16; 16	15; 16	16; 16
Standing with feet together, Foam cushion, EO	16; 16	15; 16	16; 16
Standing with feet together, Foam cushion, EC	16; 16	15; 16	16; 16
Standing with feet in tandem, EO	16; 16	15; 16	16; 16
Standing with feet in tandem, EC [b]	15; 16	14; 16	16; 16
Single-leg foam-cup stance [c]	14; 16	13; 16	15; 16

SCDS: Unilateral Superior Canal Dehiscence Syndrome group. **EO:** Eyes open. **EC:** Eyes closed.
†: In general, head IMU kinematic data was not available for 1 SCDS participant due to technical issues.
a: Standing with eyes open was not assessed in 4 healthy controls due to the scheduling constraints; therefore, no kinematic data or completion time were recorded for these participants during this task.
b: One SCDS participant was unable to perform eyes-closed tandem stance without external support; therefore, the completion time was considered zero, and no kinematic data was recorded for this participant during this trial.
c: One SCDS participant was unable to perform single-leg foam-cup stance without external support; therefore, completion time for this participant was considered zero, and no kinematic data was recorded during this trial. Additionally, single-leg foam-cup stance was not assessed in another SCDS participant due to the scheduling constraints; therefore, no kinematic data or completion time were recorded for this participant during this task.

Supplementary Table 5: Range of 3D Head Linear Accelerations

	**AP Acceleration Range *(g)***		**Lat. Acceleration Range *(g)***		**Vert. Acceleration Range *(g)***	
	SCDS	***HC***	***SCDS***	***HC***	***SCDS***	***HC***
Standing on firm surface, EO	0.141 ± 0.202	0.101 ± 0.117	0.078 ± 0.052	0.052 ± 0.027	0.108 ± 0.186	0.053 ± 0.022
Standing on firm surface, EC	0.145 ± 0.119	0.095 ± 0.039	0.108 ± 0.097	0.055 ± 0.017	0.082 ± 0.056	0.055 ± 0.021
Standing on foam cushion, EO	0.117 ± 0.047	0.096 ± 0.065	0.095 ± 0.048	0.067 ± 0.031	0.086 ± 0.043	0.065 ± 0.044
Standing on foam cushion, EC	0.219 ± 0.131	0.130 ± 0.041	0.168 ± 0.056	0.089 ± 0.027	0.189 ± 0.126	0.108 ± 0.045
Tandem stance, EO	0.165 ± 0.115	0.109 ± 0.071	0.158 ± 0.107	0.075 ± 0.027	0.136 ± 0.129	0.069 ± 0.018
Tandem stance, EC	0.176 ± 0.097	0.163 ± 0.085	0.357 ± 0.299	0.207 ± 0.244	0.220 ± 0.131	0.152 ± 0.071
Single-leg Foam-cup stance, EO	0.248 ± 0.152	0.272 ± 0.170	0.209 ± 0.163	0.169 ± 0.113	0.169 ± 0.062	0.148 ± 0.067

Values presented are means ± standard deviations. **AP:** anterior-posterior. **Lat:** lateral. **Vert:** vertical. **SCDS:** Unilateral Superior Canal Dehiscence Syndrome group. **HC:** Healthy Control group. **EO:** eyes open. **EC:** eyes closed.

Supplementary Table 6: Range of 3D Head Angular Velocities

	Yaw Velocity Range *(deg/s)*		Roll Velocity Range *(deg/s)*		Pitch Velocity Range *(deg/s)*	
	SCDS	***HC***	***SCDS***	***HC***	***SCDS***	***HC***
Standing on firm surface, EO	14.69 ± 14.15	10.37 ± 11.19	11.14 ± 11.84	7.10 ± 7.26	31.58 ± 74.61	13.04 ± 19.21
Standing on firm surface, EC	11.82 ± 6.10	7.63 ± 2.05	12.06 ± 9.60	5.81 ± 1.46	18.17 ± 18.68	9.27 ± 2.64
Standing on foam cushion, EO	15.43 ± 7.39	17.17 ± 23.43	13.61 ± 7.00	8.92 ± 4.77	18.81 ± 8.98	12.14 ± 7.75
Standing on foam cushion, EC	23.71 ± 6.16	14.30 ± 4.53	22.23 ± 8.32	12.03 ± 3.35	28.96 ± 16.07	14.31 ± 3.66
Tandem stance, EO	21.95 ± 7.94	14.19 ± 4.60	20.63 ± 11.51	9.91 ± 3.55	26.04 ± 26.29	14.08 ± 4.33
Tandem stance, EC	43.62 ± 44.51	29.57 ± 23.87	46.37 ± 34.91	23.75 ± 21.90	33.02 ± 25.57	18.64 ± 6.44
Single-leg Foam-cup stance, EO	22.75 ± 7.02	28.61 ± 22.67	28.29 ± 18.18	19.39 ± 10.96	30.14 ± 16.45	23.22 ± 10.61

Values presented are means ± standard deviations. **SCDS:** Unilateral Superior Canal Dehiscence Syndrome group. **HC:** Healthy Control group. **EO:** eyes open. **EC:** eyes closed.

Supplementary Table 7: Standard Deviation of 3D Head Linear Accelerations

	**AP Acceleration SD *(g)***		**Lat. Acceleration SD *(g)***		**Vert. Acceleration SD *(g)***	
	SCDS	***HC***	***SCDS***	***HC***	***SCDS***	***HC***
Standing on firm surface, EO	0.020 ± 0.016	0.023 ± 0.037	0.010 ± 0.005	0.008 ± 0.007	0.010 ± 0.008	0.006 ± 0.003
Standing on firm surface, EC	0.024 ± 0.019	0.016 ± 0.008	0.016 ± 0.016	0.008 ± 0.002	0.010 ± 0.006	0.008 ± 0.004
Standing on foam cushion, EO	0.020 ± 0.008	0.018 ± 0.016	0.014 ± 0.008	0.011 ± 0.007	0.010 ± 0.004	0.009 ± 0.006
Standing on foam cushion, EC	0.035 ± 0.017	0.021 ± 0.007	0.024 ± 0.009	0.013 ± 0.004	0.023 ± 0.011	0.013 ± 0.005
Tandem stance, EO	0.026 ± 0.019	0.022 ± 0.024	0.019 ± 0.011	0.011 ± 0.004	0.013 ± 0.006	0.008 ± 0.002
Tandem stance, EC	0.032 ± 0.021	0.030 ± 0.021	0.057 ± 0.060	0.024 ± 0.022	0.026 ± 0.020	0.015 ± 0.009
Single-leg Foam-cup stance, EO	0.046 ± 0.026	0.056 ± 0.042	0.026 ± 0.019	0.024 ± 0.019	0.020 ± 0.008	0.019 ± 0.011

Values presented are means ± standard deviations. **SD:** standard deviation. **AP:** anterior-posterior. **Lat:** lateral. **Vert:** vertical. **SCDS:** Unilateral Superior Canal Dehiscence Syndrome group. **HC:** Healthy Control group. **EO:** eyes open. **EC:** eyes closed.

Supplementary Table 8: Standard Deviation of 3D Head Angular Velocities

	**Yaw Velocity SD *(deg/s)***		**Roll Velocity SD *(deg/s)***		**Pitch Velocity SD *(deg/s)***	
	SCDS	***HC***	***SCDS***	***HC***	***SCDS***	***HC***
Standing on firm surface, EO	1.63 ± 1.08	1.07 ± 0.61	1.22 ± 0.90	0.75 ± 0.52	2.37 ± 3.59	1.25 ± 1.39
Standing on firm surface, EC	1.63 ± 0.77	1.06 ± 0.23	1.55 ± 1.17	0.77 ± 0.17	2.18 ± 1.64	1.20 ± 0.34
Standing on foam cushion, EO	2.19 ± 1.13	1.79 ± 1.34	1.77 ± 1.09	1.12 ± 0.40	2.21 ± 0.86	1.42 ± 0.61
Standing on foam cushion, EC	3.10 ± 0.81	2.03 ± 0.45	2.96 ± 1.18	1.58 ± 0.38	3.69 ± 1.52	2.02 ± 0.49
Tandem stance, EO	2.98 ± 1.12	1.96 ± 0.50	2.42 ± 1.31	1.30 ± 0.32	2.84 ± 1.86	1.60 ± 0.45
Tandem stance, EC	6.18 ± 5.38	3.55 ± 2.03	6.68 ± 6.14	2.71 ± 1.92	4.69 ± 3.66	2.28 ± 0.64
Single-leg Foam-cup stance, EO	3.02 ± 0.94	3.04 ± 1.70	2.99 ± 1.76	2.14 ± 0.92	3.30 ± 1.52	2.56 ± 0.93

Values presented are means ± standard deviations. **SD:** standard deviation. **SCDS:** Unilateral Superior Canal Dehiscence Syndrome group. **HC:** Healthy Control group. **EO:** eyes open. **EC:** eyes closed.

Supplementary Table 9: Range of 3D Waist Linear Accelerations

	AP Acceleration Range *(g)*		Lat. Acceleration Range *(g)*		Vert. Acceleration Range *(g)*	
	SCDS	***HC***	***SCDS***	***HC***	***SCDS***	***HC***
Standing on firm surface, EO	0.070 ± 0.031	0.047 ± 0.011	0.045 ± 0.015	0.034 ± 0.008	0.038 ± 0.023	0.026 ± 0.004
Standing on firm surface, EC	0.097 ± 0.040	0.074 ± 0.026	0.068 ± 0.028	0.044 ± 0.021	0.051 ± 0.036	0.037 ± 0.015
Standing on foam cushion, EO	0.080 ± 0.029	0.058 ± 0.014	0.077 ± 0.029	0.051 ± 0.018	0.055 ± 0.028	0.046 ± 0.017
Standing on foam cushion, EC	0.183 ± 0.080	0.106 ± 0.031	0.203 ± 0.054	0.112 ± 0.051	0.208 ± 0.123	0.105 ± 0.061
Tandem stance, EO	0.106 ± 0.042	0.077 ± 0.023	0.108 ± 0.060	0.064 ± 0.028	0.110 ± 0.066	0.056 ± 0.023
Tandem stance, EC	0.206 ± 0.086	0.135 ± 0.074	0.284 ± 0.140	0.166 ± 0.097	0.243 ± 0.091	0.160 ± 0.122
Single-leg Foam-cup stance, EO	0.220 ± 0.106	0.136 ± 0.045	0.197 ± 0.114	0.207 ± 0.093	0.158 ± 0.101	0.111 ± 0.048

Values presented are means ± standard deviations. **AP:** anterior-posterior. **Lat:** lateral. **Vert:** vertical. **SCDS:** Unilateral Superior Canal Dehiscence Syndrome group. **HC:** Healthy Control group. **EO:** eyes open. **EC:** eyes closed.

Supplementary Table 10: Range of 3D Waist Angular Velocities

	**Yaw Velocity Range *(deg/s)***		**Roll Velocity Range *(deg/s)***		**Pitch Velocity Range *(deg/s)***	
	SCDS	***HC***	***SCDS***	***HC***	***SCDS***	***HC***
Standing on firm surface, EO	9.11 ± 5.37	5.73 ± 2.19	4.96 ± 2.87	2.88 ± 1.57	8.98 ± 5.51	4.77 ± 2.16
Standing on firm surface, EC	11.06 ± 7.06	6.66 ± 2.70	7.59 ± 4.86	3.97 ± 1.64	13.55 ± 8.77	6.69 ± 2.73
Standing on foam cushion, EO	14.79 ± 9.65	9.85 ± 2.78	12.50 ± 5.85	7.21 ± 2.14	13.47 ± 7.76	8.02 ± 3.88
Standing on foam cushion, EC	26.82 ± 8.64	17.40 ± 7.08	28.30 ± 9.49	14.17 ± 5.64	33.34 ± 14.73	15.95 ± 6.53
Tandem stance, EO	22.37 ± 9.14	14.33 ± 4.40	13.11 ± 7.92	7.08 ± 2.82	20.05 ± 14.59	8.67 ± 2.41
Tandem stance, EC	44.87 ± 26.63	28.23 ± 14.16	32.49 ± 20.92	18.01 ± 11.74	48.89 ± 43.22	18.68 ± 10.28
Single-leg Foam-cup stance, EO	22.74 ± 10.56	19.06 ± 7.19	15.70 ± 8.62	13.83 ± 10.19	35.66 ± 36.15	18.40 ± 10.67

Values presented are means ± standard deviations. **SCDS:** Unilateral Superior Canal Dehiscence Syndrome group. **HC:** Healthy Control group. **EO:** eyes open. **EC:** eyes closed.

Supplementary Table 11: Standard Deviation of 3D Waist Linear Accelerations

	**AP Acceleration SD *(g)***		**Lat. Acceleration SD *(g)***		**Vert. Acceleration SD *(g)***	
	SCDS	***HC***	***SCDS***	***HC***	***SCDS***	***HC***
Standing on firm surface, EO	0.012 ± 0.005	0.008 ± 0.003	0.007 ± 0.002	0.005 ± 0.001	0.004 ± 0.002	0.004 ± 0.001
Standing on firm surface, EC	0.017 ± 0.006	0.013 ± 0.006	0.010 ± 0.004	0.006 ± 0.002	0.006 ± 0.005	0.005 ± 0.002
Standing on foam cushion, EO	0.013 ± 0.006	0.009 ± 0.003	0.010 ± 0.005	0.007 ± 0.003	0.006 ± 0.002	0.005 ± 0.002
Standing on foam cushion, EC	0.028 ± 0.009	0.017 ± 0.005	0.024 ± 0.010	0.014 ± 0.006	0.020 ± 0.010	0.011 ± 0.005
Tandem stance, EO	0.016 ± 0.006	0.013 ± 0.006	0.011 ± 0.005	0.008 ± 0.003	0.009 ± 0.004	0.006 ± 0.002
Tandem stance, EC	0.030 ± 0.014	0.020 ± 0.015	0.035 ± 0.025	0.018 ± 0.010	0.023 ± 0.011	0.014 ± 0.009
Single-leg Foam-cup stance, EO	0.044 ± 0.025	0.025 ± 0.011	0.033 ± 0.022	0.049 ± 0.031	0.015 ± 0.008	0.012 ± 0.005

Values presented are means ± standard deviations. **SD:** standard deviation. **SCDS:** Unilateral Superior Canal Dehiscence Syndrome group. **HC:** Healthy Control group. **EO:** eyes open. **EC:** eyes closed.

Supplementary Table 12: Standard Deviation of 3D Waist Angular Velocities

	Yaw Velocity SD *(deg/s)*		Roll Velocity SD *(deg/s)*		Pitch Velocity SD *(deg/s)*	
	SCDS	***HC***	***SCDS***	***HC***	***SCDS***	***HC***
Standing on firm surface, EO	1.10 ± 0.53	0.67 ± 0.25	0.55 ± 0.27	0.35 ± 0.17	0.99 ± 0.55	0.60 ± 0.24
Standing on firm surface, EC	1.32 ± 0.71	0.84 ± 0.31	0.91 ± 0.59	0.51 ± 0.19	1.54 ± 1.16	0.88 ± 0.34
Standing on foam cushion, EO	2.14 ± 1.55	1.29 ± 0.36	1.42 ± 0.69	0.93 ± 0.29	1.49 ± 0.70	0.95 ± 0.38
Standing on foam cushion, EC	3.37 ± 1.22	2.09 ± 0.71	3.35 ± 1.17	1.75 ± 0.61	3.51 ± 1.34	1.80 ± 0.62
Tandem stance, EO	3.15 ± 1.30	1.87 ± 0.45	1.38 ± 0.78	0.79 ± 0.25	1.93 ± 0.83	1.00 ± 0.28
Tandem stance, EC	6.44 ± 4.71	3.40 ± 1.68	4.13 ± 2.93	1.87 ± 1.22	4.92 ± 3.17	1.88 ± 0.91
Single-leg Foam-cup stance, EO	2.70 ± 1.11	2.23 ± 0.70	1.74 ± 0.89	1.40 ± 0.64	3.20 ± 2.46	1.78 ± 0.64

Values presented are means ± standard deviations. **SD:** standard deviation. **SCDS:** Unilateral Superior Canal Dehiscence Syndrome group. **HC:** Healthy Control group. **EO:** eyes open. **EC:** eyes closed.

Supplementary Table 13: Balance tasks completion times

	**Completion time** (maximum 30 s)		
	**HC**	**SCDS**	**p-value**
Standing on firm surface, EO	30.00 (0.00)	30.00 (0.00)	ns
Standing on firm surface, EC	29.92 (0.31)	30.00 (0.00)	ns
Standing on foam cushion, EO	29.97 (0.12)	30.00 (0.00)	ns
Standing on foam cushion, EC	30.00 (0.00)	29.94 (0.24)	ns
Tandem stance, EO	30.00 (0.00)	30.00 (0.00)	ns
Tandem stance, EC	28.68 (2.52)	15.72 (10.73)	***
Single-leg Foam-cup stance, EO	29.69 (0.61)	28.00 (7.75)	ns

Values presented are means with standard deviations in parentheses. **SCDS:** Unilateral Superior Canal Dehiscence Syndrome group. **HC:** Healthy Control group. **EO:** eyes open. **EC:** eyes closed. Significant level α= 0.05. ns: not significant. ***p-value<0.001.

Supplementary Table 14: Kinematic scores. Values reported for the kinematic scores are mean [±1SEM].

Balance Tasks	IMUs	Kinematic measures used	Healthy Controls Kinematic Score	Unilateral SCDS Group Kinematic Score	p-value
Standing on Foam EC	Waist	All 3D linear and angular ranges and SDs	70.63 [65.28, 75.97]	27.57 [20.64, 34.49]	0.0001
Standing on Foam EC	Head	All 3D linear and angular ranges and SDs	73.14 [68.65, 77.62]	29.30 [22.28, 36.33]	0.0000
Standing on Foam EC & Standing on Firm EC & Tandem Stance EO	Waist & Head	All 3D linear and angular ranges and SDs	72.79 [68.36, 77.21]	31.83 [24.95, 38.71]	0.0001

IMU: inertial measurement unit. **SCDS:** Superior Canal Dehiscence Syndrome. **SD:** standard deviation. **EO:** Eyes open. **EC:** Eyes closed. **p-value:** significance of between-group differences using an independent permutation test (α=0.05).

Supplementary Table 15: Proportion of individual SCDS kinematic scores within the thresholds defined by 1SD and 2SD of healthy group's average kinematic scores.

**Kinematic scores calculated from**	**IMUs used**	**SD of Healthy group's average kinematic score**	**% individual SCDS scores within ± 1SD of Healthy Controls score**	**% individual SCDS scores within ± 2SD of Healthy Controls score**
Standing on Foam EC	Waist	21.394	25%	43.8%
Standing on Foam EC	Head	17.367	20%	33.3%
Standing on Foam EC & Standing on Firm EC & Tandem Stance EO	Waist & Head	17.150	26.7%	40%

IMU: inertial measurement unit. **SCDS:** Superior Canal Dehiscence Syndrome. **SD:** standard deviation of the kinematic score. **EO:** Eyes open. **EC:** Eyes closed.
